# Bayesian Meta‐Learning for Few‐Shot Reaction Outcome Prediction of Asymmetric Hydrogenation of Olefins

**DOI:** 10.1002/anie.202503821

**Published:** 2025-05-02

**Authors:** Sukriti Singh, José Miguel Hernández‐Lobato

**Affiliations:** ^1^ Department of Engineering University of Cambridge Cambridge CB2 1PZ U.K.

**Keywords:** Adaptive deep kernel fitting, Asymmetric catalysis, Asymmetric hydrogenation, Bayesian, Deep kernel learning, Deep kernel transfer, Machine learning, Meta‐learning, Prototypical networks, Reaction outcome prediction

## Abstract

Recent years have witnessed the increasing application of machine learning (ML) in chemical reaction development. These ML methods, in general, require huge training set examples. The published literature has large amounts of data, but there are modelling challenges due to the sparse nature of these datasets. Herein, we report a meta‐learning workflow that can utilize the literature‐mined data and return accurate predictions with limited data. A literature dataset comprising of over 12 000 transition metal catalyzed asymmetric hydrogenation of olefins (AHO) is chosen to demonstrate the utility of our protocol. A meta‐model is trained in a binary classification setting to identify highly enantioselective AHO reactions. Two Bayesian meta‐learning approaches are considered, namely, deep kernel transfer (DKT) and adaptive deep kernel fitting (ADKF). Both these methods returned better predictions compared to prototypical network, which is another popular meta‐learning approach. Single‐task methods, such as random forest, graph neural network, and deep kernel learning, performed worse than meta‐learning methods even when trained on full training data. Additionally, we propose another meta‐learning approach called ADKF‐prior that is shown to further improve the performance in low‐data settings. The generalizability of our meta‐model is also evaluated on substrate‐ and time‐based splits. Our meta‐learning workflow can be utilized to build a pretrained meta‐model for any reaction of interest, which can then be useful to predict the outcome of new but related reactions in a few‐shot manners.

## Graphical abstract

A Bayesian meta‐learning framework is proposed for reaction outcome prediction in low‐data settings. It utilizes a literature‐mined dataset on asymmetric hydrogenation of olefins to build a pre‐trained model. This meta‐model is shown to return better predictions as compared to single‐tasks methods, such as random forest and graph neural networks, given minimal training data.

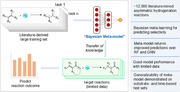

## Introduction

Catalysis is one of the fundamental principles in chemistry that plays an important role in several chemical and biological reactions. A majority of chemical products such as polymers, fuels, fine chemicals, pharmaceuticals, and so on undergo one or more catalytic transformations during their synthesis.^[^ ^1^ ^]^ Traditionally, catalyst design often involves a trial‐and‐error approach based on chemical intuition, that can be both time and resource intensive.^[^ ^2^, ^3^ ^]^ Recent years have witnessed an increasing application of machine learning (ML) approaches in catalysis.^[^ ^4^, ^5^, ^6^, ^7^ ^]^ For instance, various ML methods have been developed for reactivity and selectivity prediction.^[^ ^8^, ^9^, ^10^, ^11^ ^]^ The use of ML models for catalyst design and reaction optimization has also shown great potential.^[^ ^12^, ^13^, ^14^, ^15^ ^]^

Over the past decade, there has been significant advances in machine learning (ML) to address the challenging problems in reaction development.^[^ ^16^, ^17^, ^18^, ^19^, ^20^ ^]^ In practice, the applicability of these ML techniques is often limited as only a small amount of labeled data is available in the early stages of reaction discovery.^[^ ^21^, ^22^ ^]^ Few‐shot learning has emerged as a promising approach to handle low‐data problems.^[^ ^23^, ^24^ ^]^ It aims to train a meta‐model on a group of related tasks and generalize to predict on a new task given a few training samples. Although these methods have mainly focused on computer vision, promising results could be obtained for low‐resource molecular property prediction.^[^ ^25^, ^26^, ^27^ ^]^ Meta‐learning has also been used in molecular optimization in low‐data regimes.^[^ ^28^ ^]^ An attention‐based random forest model where the attention weights are optimized in a meta‐learning framework is reported to predict the reaction yield of a high‐throughput experimentation (HTE) dataset.^[^ ^29^ ^]^

In recent years, many meta‐learning methods have been introduced for the few‐shot learning setting. Primarily, there are two approaches to meta‐learning. The first approach is metric‐based meta‐learning, which includes models like Siamese neural networks, matching networks, prototypical networks, relation networks, and so on.^[^ ^30^, ^31^, ^32^, ^33^ ^]^ The second approach is optimization‐based meta‐learning with model‐agnostic meta‐learning (MAML) as one of the popular representative ideas that has inspired more meta‐learning algorithms.^[^ ^34^ ^]^ Previous works have also proposed Bayesian meta‐learning methods that combines deep networks with probabilistic methods.^[^ ^35^, ^36^, ^37^ ^]^ These methods are designed solely for neural networks (NNs), which generally lack reliable uncertainty estimates.^[^ ^38^ ^]^

In many real‐world problems, the uncertainty quantification in addition to accurate predictions enhance the usability of an ML model. For instance, in reaction development, Bayesian optimization (BO) can utilize the uncertainty estimates to guide future experiments in search of optimal reaction conditions.^[^ ^39^ ^]^ While there are several approaches to quantify uncertainty, Gaussian processes (GPs) are generally preferable for small datasets.^[^ ^40^ ^]^ However, training GPs on complex and high dimensional molecular data is challenging. Deep kernel learning (DKL) provides an interesting framework by combining the representational flexibility of NNs with the uncertainty estimates of GPs.^[^ ^41^ ^]^ DKL has shown promise in various molecular property and reaction outcome prediction tasks.^[^ ^42^, ^43^ ^]^ However, DKL can lead to overfitting in case of small datasets.^[^ ^44^ ^]^ Subsequently, the meta‐learning framework has been used for fitting deep kernel GPs to a distribution of datasets. Deep kernel transfer (DKT) and adaptive deep kernel fitting with implicit function Theorem (ADKF‐IFT) are important examples.^[^ ^45^, ^46^ ^]^

In this work, we present a meta‐learning framework for reaction outcome prediction. Although ML models have shown great success in reaction outcome prediction on well‐standardized HTE datasets, it remains challenging to model literature‐derived datasets.^[^ ^47^ ^]^ To demonstrate the utility of our meta‐learning protocol, a literature‐mined dataset based on transition metal catalyzed asymmetric hydrogenation of olefins (AHO) is chosen. We show that our meta‐learning model can provide improved predictions on the AHO dataset. Additionally, we report another meta‐learning approach for training deep kernel GPs specifically suited for problems in low‐data regime. The ability of our meta‐model to generalize and make accurate predictions with small amounts of data is shown using an out‐of‐sample test set. This can be useful during early stages of reaction development for predicting the reaction outcome when only minimal data is available.

## Results and Discussion

**Figure 1:**
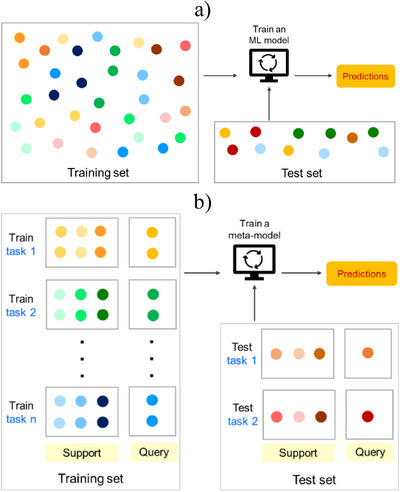
A diagram illustrating a) conventional machine learning and b) meta‐learning methods. The conventional approach requires a single large dataset to train the ML model and obtain predictions on the test set. Meta‐learning involves training on a set of related tasks such that the meta‐model can generalize and provide accurate predictions on the test task with limited number of examples.

The conventional supervised methods utilize a large, labeled dataset D to train an ML model (Figure 1a). A held‐out test set is then used for model evaluation. Meta‐learning, on the other hand, involves training a meta‐learner on a set of related supervised tasks, such that it can rapidly generalize to a new task, given limited examples (Figure 1b).^[^ ^48^ ^]^ Specifically, we have a set of training tasks $D_{\mathrm{train}}={\left\{T_{t}\right\}}_{t=1}^{N}$ and each task $T_{t}={\left\{\left(x_{i},y_{i}\right)\right\}}_{i=1}^{N_{T}}$ is comprised of a support set $S_{T}$, used to train the model and a query set $Q_{T}$, for evaluation of the model performance. During the meta‐training phase, the model is trained on multiple tasks sampled from $D_{train}$ so as to accurately predict the labels of the query set, given the features and labels of the support set. At meta‐testing time, the performance of the meta‐learner is finally evaluated on the query set ${Q_{T}}_{*}$, given a small support set ${S_{T}}_{*}$ associated with an unseen task $T_{*}$ sampled from the test set.

**Figure 2:**
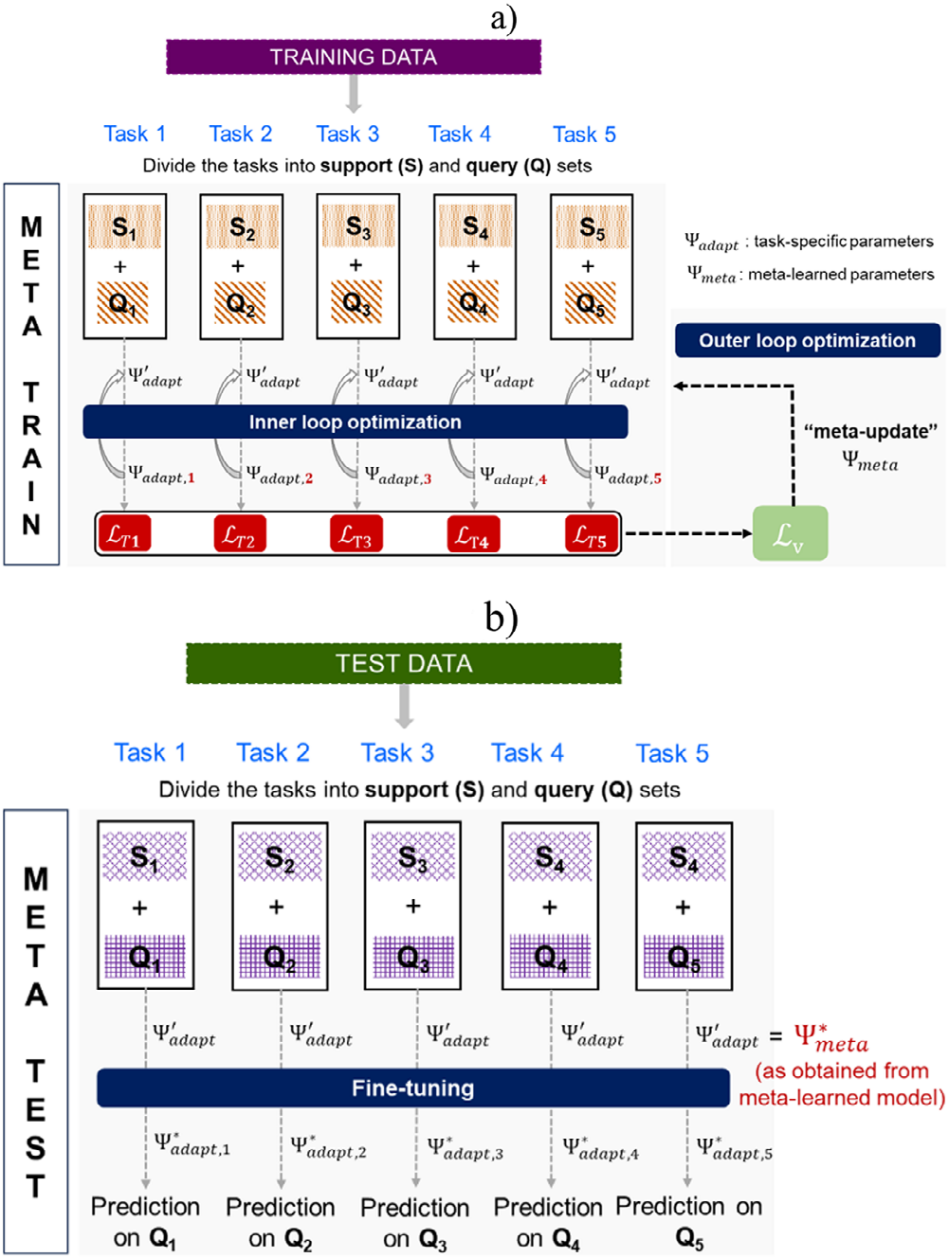
A general schematic representation of the hierarchical model of meta‐learning. a) During meta‐training time, the task‐specific parameters Ψ_*adapt*_ are adapted to individual tasks by minimizing the training loss $L_{T}$ on the support set. A set of meta‐parameters Ψ_*meta*_ shared among tasks make this adaptation more efficient and are typically used to initialize task‐specific parameters Ψ_*adapt*_. The meta‐parameters are updated by optimizing the expected validation loss $L_{V}$. b) During meta‐test time, the task‐specific parameters Ψ_*adapt*_ are initialized using the optimal meta‐parameters $\Psi_{meta}^{*}$ and fine‐tuned on individual tasks to obtain the predictions on the query set.

Meta‐learning has also been interpreted as a hierarchical model (Figure 2).^[^ ^49^ ^]^ It considers two sets of parameters. First, a set of task‐specific parameters Ψ_*adapt*_ are optimized in an inner loop. Second, a set of meta (or task common) parameters Ψ_*meta*_ are optimized in an outer loop, given the optimized task‐specific parameters (Figure 2a). During the meta‐training phase, task‐specific parameters are adapted to each task to get the best possible model on the support set for the given value of meta‐parameters. The meta‐parameters are learned to obtain the best possible average validation loss (on the query set) over multiple training tasks (Figure 2a). Specifically, Ψ_*adapt*_ is learned by minimizing the training loss $L_{T}$ on the task's support set $S_{T}$. The meta‐parameters Ψ_*meta*_ are optimized by minimizing the expected validation loss $L_{V}$ on a set of training tasks. During meta‐testing, the optimized meta‐parameters $\Psi_{meta}^{*}$ provide an initialization to the task‐specific parameters Ψ_*adapt*_ (Figure 2b). These task‐specific parameters can then be fine‐tuned on the support set to obtain predictions on the query set.

**Figure 3:**
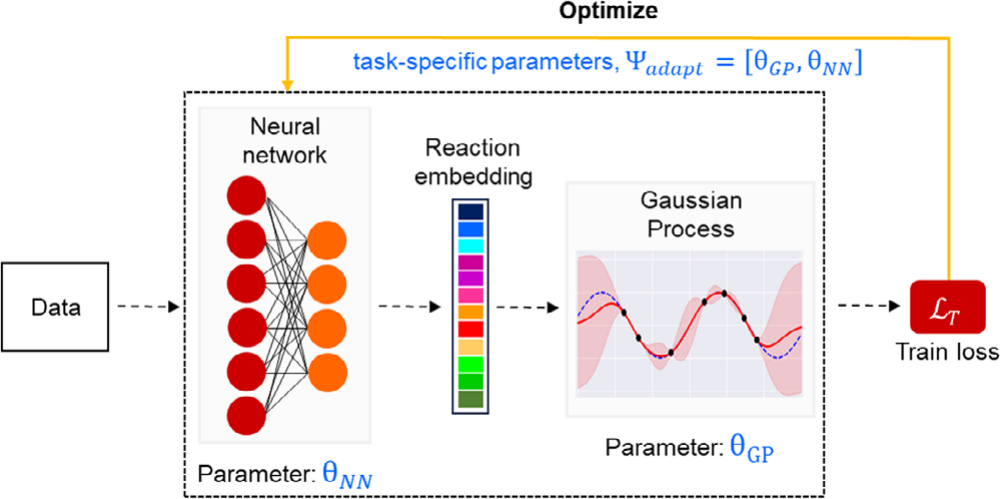
A schematic diagram of deep kernel learning. A neural network parameterized by θ_*NN*_ is used as a feature extractor. The reaction embedding thus obtained is passed to a Gaussian process parameterized by θ_GP_ to obtain predictions with uncertainty estimates.

In this work, we have considered two Bayesian meta‐learning methods: deep kernel transfer (DKT)^[^ ^46^ ^]^ and adaptive deep kernel fitting (ADKF).^[^ ^47^ ^]^ Unlike many meta‐learning methods, DKT and ADKF can work on both classification and regression settings. Both approaches provide a novel framework for fitting a deep kernel to a distribution of tasks. Deep kernels are a combination of neural networks with kernels that provide expressive and scalable covariance functions for Gaussian process models.^[^ ^50^ ^]^ In deep kernel learning (DKL),^[^ ^41^ ^]^ a neural network $f_{\theta_{\mathrm{NN}}}$ (with parameters θ_*NN*_) is used to map an input vector *x* to a latent vector $z\;=f_{\theta_{\mathrm{NN}}}\;\left(x\right)$. These latent embeddings are then passed as an input to the kernel k(*z*, *z*′|θ_GP_) parameterized by θ_GP_ (Figure 3). Therefore, the kernel can be constructed as $k\;\left(z,z^{\prime }|\theta_{\mathrm{GP}},\theta_{\mathrm{NN}}\right)=k^{\prime }\;\left(f_{\theta_{\mathrm{NN}}}\left(x\right),f_{\theta_{\mathrm{NN}}}\left(x^{\prime }\right)|\theta_{\mathrm{GP}}\right)$. The parameters of the deep kernel, that is, (θ_GP_,θ_NN_) are learned jointly by minimizing the negative log marginal likelihood of a Gaussian process model on a single dataset. DKT and ADKF differ from DKL in the fact that both use meta‐learning to train the deep kernel Gaussian processes.

**Figure 4:**
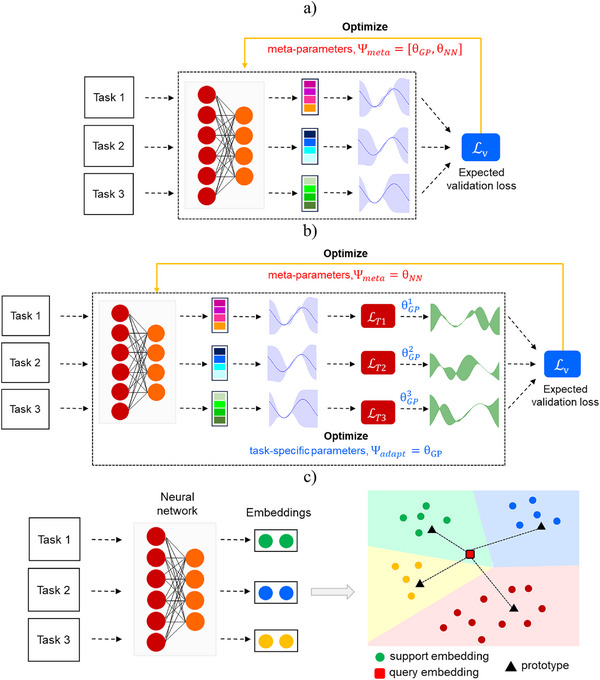
An overview of the meta‐learning methods employed in this study: a) deep kernel transfer (DKT), b) adaptive deep kernel fitting (ADKF), and c) prototypical networks. While prototypical network is a metric‐based meta‐learning approach, DKT and ADKF are based on Bayesian approach to train deep kernel Gaussian processes.

DKT jointly optimizes the neural network parameters θ_NN_ and base kernel parameters θ_GP_ by minimizing the expected negative marginal log likelihood across all the available data in the training tasks (Figure 4a). DKT makes the assumption that different tasks can be well‐approximated using identical hyperparameters for the base kernel on each task. ADKF, on the other hand, adapts the base kernel parameters θ_*GP*_ individually to each task (Figure 4b). In ADKF, the base kernel parameters are learned by optimizing the negative log marginal likelihood computed on the support set. The neural network parameters θ_*NN*_ are meta‐learned across tasks by evaluating the negative log joint predictive posterior on the query set given the support set. Furthermore, both DKT and ADKF provide predictions with corresponding uncertainty estimates that might be useful, for instance, in BO to guide the search for optimal reaction conditions.

As a baseline for the methods described above, we have also considered another popular meta‐learning approach, namely, prototypical networks. The prototypical network is a metric‐based meta‐learning method and works only for classification settings (Figure 4c).^[^ ^29^ ^]^ First, a neural network is used to map the input into an embedding space. The prototype is computed as the mean of support set embeddings belonging to its class. The embedded query sample is then classified by locating the nearest class prototype.

## Dataset Details

A previous study reported a literature‐mined dataset based on transition‐metal‐catalyzed asymmetric hydrogenation of olefins (AHO).^[^ ^51^ ^]^ The dataset consists of ∼12 000 transition‐metal‐catalyzed AHO reactions. In this work, we utilize the Ir‐ and Rh‐catalyzed AHO reactions to build a meta‐model that can predict the selectivity of new AHO reactions with limited training data (Section 1 in the Supporting Information). These new AHO reactions can have substrates or ligands that are unseen in the training data. The reaction performance in terms of enantioselectivity is skewed toward highly selective reactions for both metal catalysts. Therefore, we formulate the enantioselectivity prediction as a binary classification problem.

In a recent work, we provided a detailed data‐driven analysis of the AHO dataset.^[^ ^52^ ^]^ The type of olefins, ligands, reaction conditions etc. used with different metal catalysts are analyzed together to understand the reactivity patterns. The type of olefins in this dataset belongs to six categories: aryl‐ and alkyl‐substituted olefins, enols, enamides, and enamines, allylic alcohol and ether, α,β‐unsaturated carbonyls, and alkenes bearing heteroatoms such as Si, B, P, etc. The asymmetric hydrogenation with Ir catalyst is dominated by aryl‐ and alkyl‐substituted olefins, whereas, α,β‐unsaturated carbonyls and enamides are primary olefin types with Rh‐catalyzed AHO reactions. The type of ligands is primarily P,N ligands for Ir catalyst with phosphine P donor and oxazoline N donor been most widely used. For Rh catalysts, mono‐ and bidentate phosphorous ligands are most common with phosphoramidites and bisphosphines used in 70% of the reactions. Also, 80% of the Ir‐catalyzed asymmetric hydrogenation reactions are reported with dichloromethane as the solvent. For Rh‐catalyzed asymmetric hydrogenation, 40% of reactions are reported with dichloromethane and remaining 60% use polar coordinating solvents such as methanol, tetrahydrofuran, etc. Additionally, the sparsity of data with respect to the experimentally available olefin‐ligand combinations is highlighted as well. Although the dataset contains a large number of different olefins and ligands, the majority of the reported reactions are comprised of only a few olefin‐ligand combinations. This highlights the challenge in modeling literature datasets using machine learning algorithms. In the next section, we describe our meta‐learning approach and compare its predictive ability with standard ML methods.

We apply the meta‐learning method in a binary classification setting to predict the enantioselectivity (%ee) of the AHO reaction. The data is divided into two classes, that is, reactions with %ee (a) greater than 80 and (b) less than 80. The resulting classes have 65% and 35% examples respectively. The area under the precision‐recall curve (AUPRC) score is used to evaluate the model performance, which is sensitive to class imbalance. Next, the important step of molecular featurization is carried out. The AHO reaction comprises of several chemical entities. Of these, the olefins, ligands, and solvents are featurized using the Morgan fingerprints as 512‐bit vector with radius 2.^[^ ^53^ ^]^ One‐hot encoding is used to include the identity of metal and presence/absence of additive in the reaction featurization. Furthermore, the reaction condition comprising of temperature, pressure, and catalyst loading is considered as well. The concatenation of fingerprints, one‐hot encodings, and reaction condition provides the full reaction representation, with a dimension of 1544. This is then used as an input to the meta‐learning and single‐task models. The only exception is graph neural network where molecular graph is used as the input representation. A message passing neural network is then used to learn the feature embeddings (Section 2 in the Supporting Information). The graph embeddings of olefins, ligands, and solvents are concatenated with other features to obtain the reaction representation of size 1544.

The meta‐training starts with partitioning the training tasks into support and query. The support and query set size is chosen to be 512 and 64, respectively. The validation tasks are used to optimize the hyperparameters of the meta‐model (Section 3 in the Supporting Information). During training, the support sets in a batch of five training tasks are iteratively fed to the meta‐learner. The average prediction log‐likelihood on corresponding query sets is maximized for these training tasks. During meta‐testing time, the test task is further divided into support and query sets (Figure 2b). The meta‐trained model is fine‐tuned on the support set of the test task to obtain predictions on the corresponding query set. For an unbiased evaluation, we report performance averaged over multiple support‐query splits of the test task. In this work, we use support set of varying sizes to show the utility of meta‐model for low‐data prediction. Also, we compare the performance of meta‐learning models with other popular single‐task methods: random forest (RF), graph neural network (GNN), deep kernel learning, decision trees, extreme gradient boosting (XGBoost), adaptive boosting (AdaBoost), ExtraTrees, and support vector machine (SVM). For a suitable comparison, the performance of single‐task methods trained on full training data $D_{train}$ is reported.

Meta‐learning requires a large set of training tasks for pretraining and a set of test tasks. There can be many ways of splitting the dataset into training and test tasks. The most common approach to evaluate reaction outcome prediction models is using random train‐test splits. However, in practice we often require the model to return meaningful predictions on test set containing, for instance, substrates different than that used in training. Herein, we consider three different methods to construct tasks for meta‐learning: random splits, substrate‐based splits, and time‐based splits. The performance of meta‐learning and single‐task methods are then evaluated on all three data splits.

## Meta‐Learning with Random Splits

**Figure 5:**
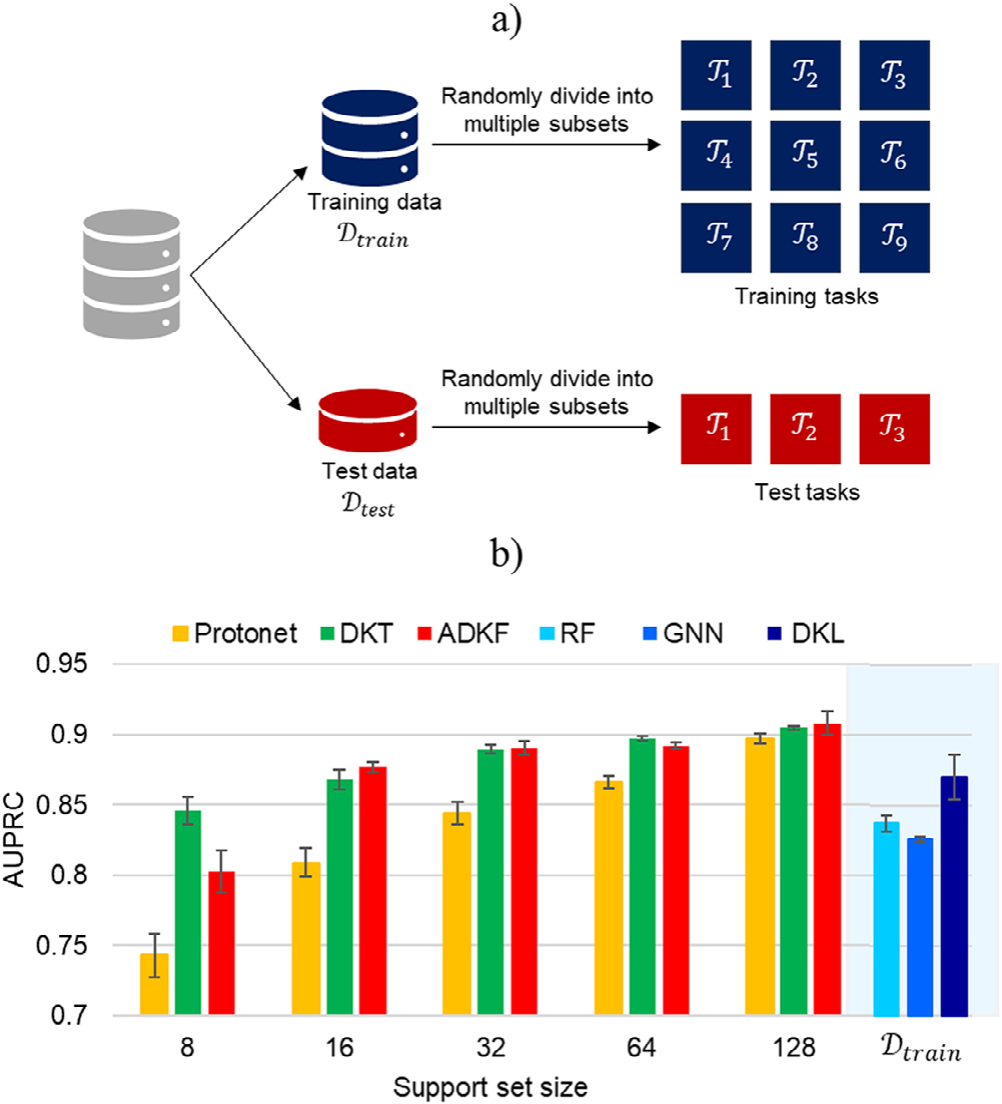
a) Construction of tasks for meta‐learning by randomly splitting the dataset into multiple subsets, with each subset representing a different task. b) A comparison of model performance on random splits in terms of AUPRC score for meta‐learning and single‐task methods. The performance of meta‐learning methods is reported for five different support set sizes. For a suitable comparison, the performance of single‐task methods is reported with $D_{train}$ as the training set. The error is reported as standard deviation across 10 runs.

The dataset D is first partitioned into 80:20 training $D_{train}$ and test $D_{test}$ sets. A small fraction of training data is used for validation $D_{valid}$. To construct the tasks for meta‐learning, the training set $D_{train}$ is randomly divided into multiple subsets of the data, where each subset corresponds to a different training task (Figure 5a). The test and validation tasks are obtained in the same manner. At meta‐test time, the model performance averaged over 10 random support‐query splits of each test task is reported. The error is reported as standard deviation across these 10 runs. To analyze the model performance with varying amount of training data available for each test task, five different support set sizes are considered: 8, 16, 32, 64, and 128. A query set size of 128 is used with all support set sizes.

A summary of results for various meta‐learning and single‐task methods is presented in Figure 5b. Among meta‐learning methods, DKT and ADKF outperform prototypical network (Protonet) for all support set sizes. The difference in performance is more pronounced for smaller support sets. An improvement in performance is observed for all meta‐learning methods with an increase in support set examples of test tasks. To get a reasonable comparison, the performance of single‐task methods is obtained by utilizing $D_{train}$ for model training (Section 4 in the Supporting Information). Out of all single‐task methods, DKL performs significantly better than RF and GNNs. Also, the performance of DKL trained on full training data $D_{train}$ is comparable to meta‐learning methods (DKT and ADKF) with a support set consisting of only 16 examples. The performance of single‐task methods is provided in Table 4.2 of the Supporting Information.

Since DKT and ADKF perform better than Protonet (Figure 5b), we wondered if predictions can further be improved, especially in low‐data settings. In ADKF, only GP hyperparameters θ_*GP*_ are optimized in the inner loop optimization (Figure 4b). The optimization of neural network parameters θ_*NN*_ in the inner loop might result in overfitting owing to large number of parameters. To overcome this issue, we propose a meta‐learning approach (ADKF‐prior) that assigns a Gaussian prior over the neural network parameters θ_NN_. The feature extractor is adapted for each task according to this meta‐learned prior in such a way that small deviations are allowed across tasks. The prior can provide a good initialization for θ_*NN*_, which is then fine‐tuned on individual tasks. This would likely prevent over‐fitting when only small amount of data is available. In the next section, we describe the details of our ADKF‐prior method.

## ADKF with Gaussian Prior

**Figure 6:**
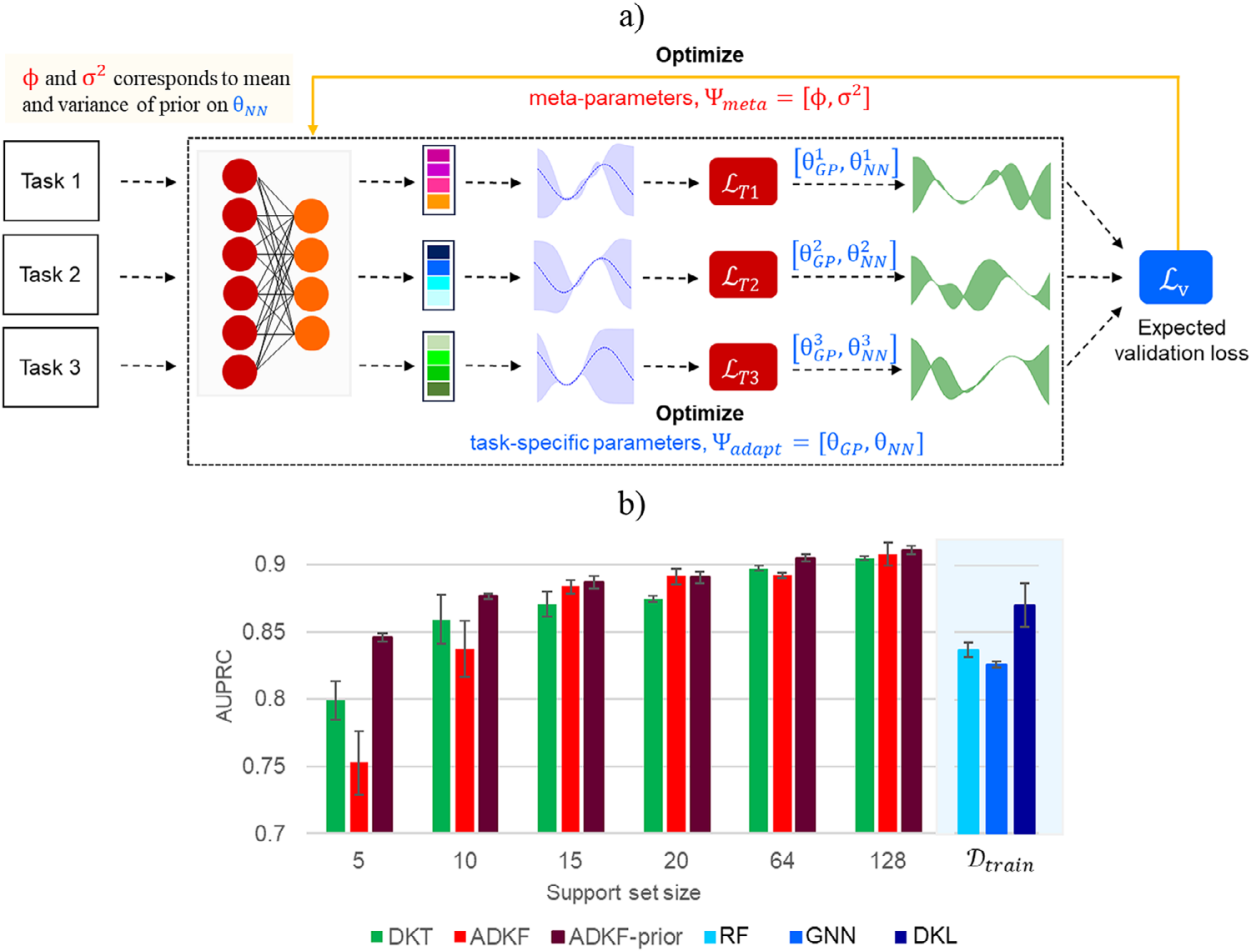
a) A diagram illustrating the ADKF‐prior approach to train deep kernel Gaussian processes with meta‐learning. b) Summary of model performance in terms of AUPRC scores of different Bayesian meta‐learning methods with varying support set sizes. For a suitable comparison, the performance of single‐task methods is reported with $D_{train}$ as the training set. The error is reported as standard deviation across 10 runs.

Let θ_*GP*_ and θ_*NN*_ denote the parameters of base kernel and feature extractor, respectively. We consider task‐specific parameters Ψ_*adapt*_ = [θ_*GP*_,θ_*NN*_] to be the set of all parameters in a deep kernel. Since the size of support set is usually small and the feature extractor has a large number of parameters, some form of regularization is required for efficient learning while preventing overfitting in the inner loop. We incorporate this by employing a hierarchical Bayesian model and assigning a Gaussian prior to the feature extractor parameters (Figure 6a). Thus, $\theta_{\mathrm{NN}}∼N\left(\theta_{\mathrm{NN}}|\phi ,\sigma^{2}I\right)$ is normally distributed with mean ϕ and variance σ^2^. The estimation of these prior parameters corresponds to meta‐learning, with Ψ_*meta*_ = [ϕ, σ^2^].

During meta‐training, the task‐specific parameters Ψ_adapt_ are estimated by minimizing the train loss $L_{T}$ on the support set $S_{T}$, given the meta‐learned parameters Ψ_meta_. The optimal task‐specific parameters $\Psi_{adapt}^{*}$ are obtained by solving the inner loop optimization problem with gradient‐based methods. Using $\Psi_{\mathrm{adapt}}^{*}$, the meta‐learned parameters Ψ_*meta*_ are estimated in the outer loop optimization by minimizing the average predictive validation loss $L_{V}$ on the query set $Q_{T}$ of T randomly sampled training tasks. During meta‐testing, the predictive posterior distribution is used to make predictions on the test task $T_{*}$ with the optimal parameters $\Psi_{\mathrm{adapt}}^{*}$ and $\Psi_{\mathrm{meta}}^{*}$ obtained after meta‐training. For more details see Sections 5 and 6 in the Supporting Information.

Next, we compare the performance of ADKF‐prior with two other Bayesian meta‐learning approaches, that is, DKT and ADKF. To display the utility of ADKF‐prior in low data settings, four different support set sizes of 5, 10, 15, and 20 are used. Additionally, slightly larger support sets of size 64 and 128 are also considered. The query set size of 128 is constant across different support sets. A comparison of model performance of all three Bayesian meta‐learning approaches in terms of AUPRC scores is shown in Figure 6b. The results are reported as an average over 10 different support‐query splits of the test tasks. The error is reported as standard deviation across these 10 runs. It can be noted from Figure 6b that ADKF‐prior significantly outperforms DKT and ADKF with smaller support sets of size 5 and 10. Also, the model performance improves with an increase in number of examples in the support set. All three methods provide comparable performance for larger support sets, for instance, support set size of 128 (Section 7 in the Supporting Information).

## Meta‐Learning with Substrate‐Based Splits

**Figure 7:**
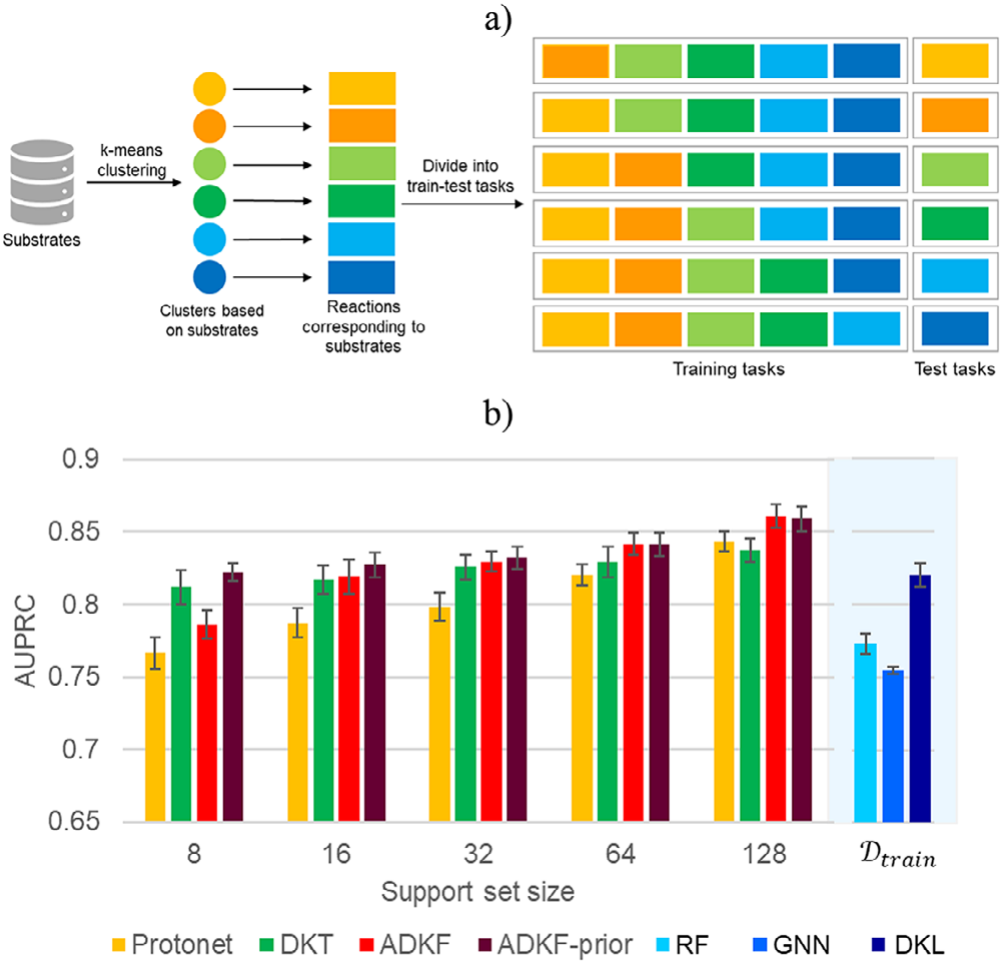
a) Construction of tasks for meta‐learning by using substrate‐based splits. Substrates are first grouped into different clusters. The reactions corresponding to each substrate cluster are identified. One cluster is kept as test task while remaining clusters are used as training tasks. b) A comparison of model performance on substrate‐based splits in terms of AUPRC score for meta‐learning and single‐task methods. The performance of meta‐learning methods with standard errors is reported for five different support set sizes. For a suitable comparison, the performance of single‐task methods is reported with $D_{train}$ as the training set.

Given the better performance of meta‐learning over single‐task algorithms on random splits, we decided to evaluate these methods on more challenging test tasks. For this purpose, we use substrate‐based splits where the substrates in the test task are different than those present in the training tasks. The dataset contains over 2400 unique olefin substrates. The substrates are first featurized using 166‐bit 2D structural fingerprints, known as molecular access system keys.^[^ ^54^ ^]^ The uniform manifold approximation and projection (UMAP) plot is used to reduce the feature dimensions to two.^[^ ^55^ ^]^ This is followed by k‐means clustering to identify the substrate clusters. A leave‐one‐cluster‐out (LOCO) type approach is used to construct the tasks for meta‐learning (Figure 7a). From a total of six clusters, one cluster is kept as a test task while remaining five clusters form the training tasks. In this way, a combination of six train‐test tasks is obtained where each cluster is used as test task at least once (Figure 7a). Having access to the trained meta‐model, it is straightforward to predict the likely outcome of reactions with a new substrate, given only a limited number of related training examples.

The performance of all four meta‐learning methods is then evaluated on substrate‐based splits. Here, we describe the results for one of the train‐test task combinations (Figure 7a), while the remaining five are reported in Section 8 of the Supporting Information. A summary of model performance is shown in Figure 7b. The model is evaluated on five different support set sizes: 8, 16, 32, 64, and 128. The query set size of the test task is kept at 128. The final performance is reported as an average over 30 support‐query splits of the test task. It can be noted from Figure 7b that all three Bayesian meta‐learning methods perform better than prototypical networks, particularly with low support set sizes. An improvement in model performance is observed with an increase in the support set size of the test task. Among the Bayesian meta‐learning methods, DKT returns better predictions than ADKF with support set sizes of 8 and 16. With greater support set sizes of 64 and 128, ADKF performs better than DKT. On the other hand, ADKF‐prior shows good predictive ability with all support set sizes (Figure 7b). We also compared the performance of meta‐learning approaches with single‐task methods. The single‐task methods are trained on full training data $D_{train}$. The results for RF, GNN, and DKL are shown in Figure 7b, while it is provided in Section 8.1.2 of the Supporting Information for all other single‐task methods. Among the single‐task methods, DKL provides the best predictive performance. When compared with meta‐learning methods, DKL has performance similar to ADKF‐prior with only 8 examples in the support set of the test task (Figure 7b). We also evaluated the performance of single ‐task methods trained on $D_{train}$+ support set of the test task. No considerable change in model performance is noted except for DKL, which shows a slight improvement (Table S7 in the Supporting Information). These results indicate the utility of our meta‐learning approach to generalize to unseen substrate types. Additionally, we have also evaluated the model performance on two other classification thresholds: 85 and 90 (Section 8.1.3 in the Supporting Information). It is noted that the meta‐learning methods obtained better predictive performance compared to single‐task methods.

## Meta‐Learning with Time‐Based Splits

To further demonstrate the applicability of our meta‐learning method, we evaluate the model performance on time‐based splits (Section 9 in the Supporting Information). The AHO dataset contains reactions published between 2000–2020. These are used for training the meta‐model. We selected an additional set of 245 Ir‐ and Rh‐catalyzed AHO reactions reported between 2023–2024.^[^ ^56^, ^57^, ^58^, ^59^, ^60^ ^]^ These reactions form the test task to evaluate the performance of meta‐learning methods. The meta‐model trained on the full AHO dataset is used to obtain predictions on the new test task. Four different support set sizes of 8, 16, 32, and 64 are considered, while query set size remains 128. The final model performance is reported as an average over 10 random support and query splits of the test tasks. The error is reported as standard error across these 10 runs.

**Figure 8:**
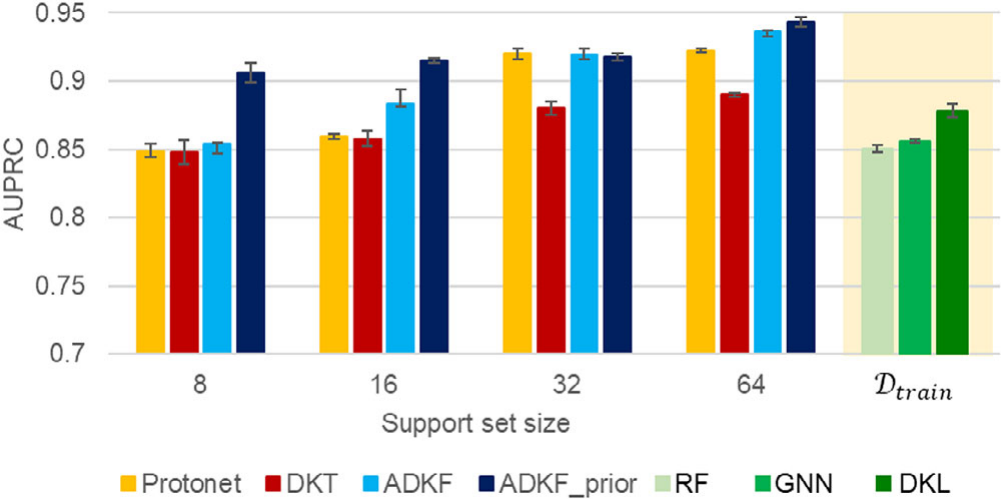
A comparison of the performance of different meta‐learning and single‐task methods on time‐based splits. The error is reported as standard error across 10 runs.

We compare the performance of all four meta‐learning methods. A summary of results is presented in Figure 8. It can be noted from Figure 8 that ADKF‐prior performs significantly better as compared to other three methods with lower support set sizes of 8 and 16. In case of relatively larger support sets of size 32 and 64, ADKF‐prior has a comparable performance to that of ADKF. We also compared the performance with single‐task methods. The single‐task methods are trained using the full training data $D_{train}$. Out of three single‐task methods, the best performance with an AUPRC score of 0.8782 ± 0.0150 is obtained with DKL, while RF and GNN yielded an AUPRC score of 0.8504 ± 0.0077 and 0.8558 ± 0.0042, respectively. Thus, the performance of single‐task methods trained on $D_{train}$ is comparable to meta‐learning methods with only eight examples in the support set. Also, ADKF‐prior with an AUPRC score of 0.9062 ± 0.0221 with support set size of eight, significantly outperforms other meta‐learning and single‐task methods. These results signify the importance of meta‐learning approaches for reaction outcome prediction when only limited data is available.

## Conclusion

Meta‐learning can have great potential in enabling the use of large amount of literature data for reaction outcome prediction. In particular, a meta‐model pretrained on any reaction of interest can provide initial estimates on the success (in terms of yield and/or enantioselectivity) of new but related reactions with limited data. In this work, we have utilized a large literature‐mined dataset based on transition metal‐catalyzed asymmetric hydrogenation of olefins (AHO). Various meta‐learning approaches are used to predict the enantioselectivity of the AHO reaction. A meta‐model is first trained on a set of diverse training tasks such that it can efficiently adapt to unseen test tasks. Two Bayesian meta‐learning approaches, that is, deep kernel transfer (DKT) and adaptive deep kernel fitting (ADKF) are employed. Prototypical networks based on metric‐based meta learning are used as a baseline. In addition, three popular single‐task methods: random forests, graph neural network, and deep kernel learning are used for performance comparison. We report the model performance with varying amount of training data available in test tasks. The meta‐learning methods significantly outperform the single‐task methods. Also, the Bayesian meta‐learning methods return better prediction than prototypical networks. Based on this observation, we proposed another Bayesian meta‐learning approach namely, ADKF‐prior, that further improves the model performance, especially when very small amount of training data is available for the test task. The generalizability of our meta‐model is evaluated on substrate‐based and time‐based splits. Among single task methods, the best performance is observed with deep kernel learning utilizing full training data. In the case of meta‐learning methods, ADKF‐prior with only eight training examples in the test task performs better than single‐task methods. ADKF‐prior also outperforms other meta‐learning methods in low‐data settings. With more examples, all four meta‐learning methods obtained comparable performance. These findings suggest that our meta‐learning protocol can be advantageous in initial phase of reaction development when minimal data is available.

## Supporting Information

Additional figures, tables and technical details are provided in the Supporting Information. The code is publicly available through https://github.com/sukriti243/Bayesian‐meta‐learning‐for‐reaction‐outcome‐prediction.

## Conflict of Interests

The authors declare no conflict of interest.

## Supporting information



Supplementary Information

## Data Availability

The data that support the findings of this study are available from Xin Hong (author of “Angew. Chem. Int. Ed. 2021, 60, 22804–22811”). Restrictions apply to the availability of these data, which were used under license for this study. Data are available at https://asymcatml.net/ with the permission of Xin Hong (author of “Angew. Chem. Int. Ed. 2021, 60, 22804–22811”).
