# Supplementary material for: Bayesian Meta‐Learning for Few‐Shot Reaction Outcome Prediction of Asymmetric Hydrogenation of Olefins
Source: Angew Chem Int Ed Engl. 2025 May 2;64(27):e202503821. doi: 10.1002/anie.202503821 (PMC12207359; doi:10.1002/anie.202503821)
Supplement: Supplementary file 1 — Supplementary Information [file ANIE-64-e202503821-s001.pdf]

Supporting Information for

Bayesian Meta-Learning for Few-Shot Reaction Outcome Prediction of Asymmetric Hydrogenation of Olefins

Sukriti Singh* and José Miguel Hernández-Lobato*

Department of Engineering, University of Cambridge, Cambridge CB2 1PZ, U.K.

sukriti243@gmail.com; jmh233@cam.ac.uk

Section	Table of Contents	Page No.
1.	Reaction details	S3
2.	Feature representation	S3
3.	Implementation	S4
	3.1 Single-task methods	S4
	3.2 Meta-learning methods	S6
4.	Model performance on random splits	S7
	4.1 Performance of meta-learning methods	S7
	4.2 Performance of single-task methods	S8
5.	Mathematical details of ADKF-prior	S9
6.	Implementation of ADKF-prior	S12
7.	Performance comparison of DKT and ADKF with ADKF-prior on random splits	S13
8.	Model performance on substrate-based splits	S13
	8.1 Train-test split 1	S14
	8.1.1 Performance of meta-learning methods	S14
	8.1.2 Performance of single-task methods	S15
	8.1.3 Performance with different classification thresholds	S16
	8.2 Train-test split 2	S18
	8.2.1 Performance of meta-learning methods	S18
	8.2.2 Performance of single-task methods	S19
	8.3 Train-test split 3	S21
	8.3.1 Performance of meta-learning methods	S21
	8.3.2 Performance of single-task methods	S22
	8.4 Train-test split 4	S23
	8.4.1 Performance of meta-learning methods	S23
	8.4.2 Performance of single-task methods	S24
	8.5 Train-test split 5	S25
	8.5.1 Performance of meta-learning methods	S25
	8.5.2 Performance of single-task methods	S26
	8.6 Train-test split 6	S27
	8.6.1 Performance of meta-learning methods	S27
	8.6.2 Performance of single-task methods	S28
9.	Performance on out-of-sample test set	S30
10.	References	S32

1. Reaction details

To demonstrate our meta-learning workflow, we use a literature-mined dataset based on transition-metal-catalyzed asymmetric hydrogenation of olefins (AHO). The dataset is available through http://asymcatml.net (accessed 2023-08-13). The access to the database is provided by registration on the website followed by signing a user license agreement. The AHO dataset consists of ~12000 reactions catalyzed by Ir and Rh metal catalysts (Figure S1). The dataset contains 5009 Ir-catalyzed AHO reaction with 1181 and 805 unique olefins and ligands respectively. There are 6391 Rh-catalyzed AHO reactions with 1386 and 721 distinct olefins and ligands respectively. The reaction performance in terms of enantioselectively is skewed towards highly selective reactions for all three metal catalysts.

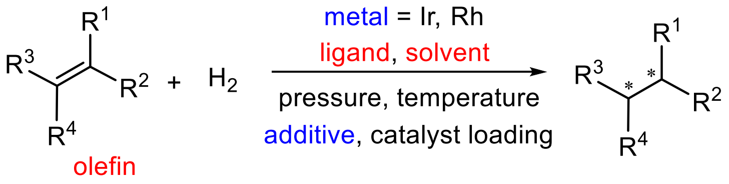

Figure S1. A general scheme for asymmetric hydrogenation of olefins.

The type of olefins in this dataset belongs to six categories: aryl- and alkyl-substituted olefins, enols, enamides and enamines, allylic alcohol and ether, α,β-unsaturated carbonyls, and alkenes bearing heteroatoms such as Si, B, P, etc. The type of ligands are primarily P,N ligands for Ir and bisphosphines for both Rh and Co metal catalysts.

2. Feature representation

The reaction consists of both categorical and continuous variables (Figure S1). While continuous variables can be incorporated directly, the categorical variables need to be featurized into continuous representation. The categorical and continuous reaction components considered in this work are as following:

(1) Categorical features: olefin, ligand, solvent, metal, additive. Of these, olefin, ligand, and solvent are featurized using Morgan fingerprints of dimension 512 and radius 2. Whereas, the identity of metal catalysts and presence/absence of additive are one-hot encoded (Figure S2).

(2) Continuous features: temperature, pressure, catalyst loading

The final reaction representation is a result of concatenation of the feature representations of all individual components, providing a 1544-dimensional vector (Figure S2).

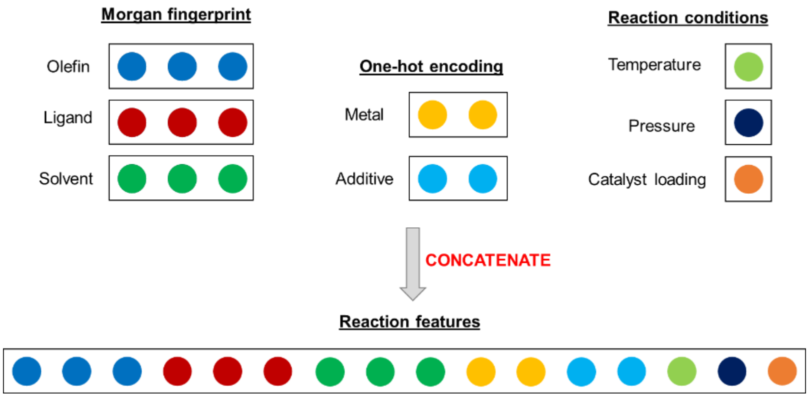

Figure S2. Reaction representation by concatenating the features of individual reaction components.

Additionally, we have also molecular graphs of olefins, ligands, and solvents to obtain the reaction representation for graph neural networks. Each of the molecule is presented as an undirected molecular graph with atoms as nodes and bonds as edges. The nodes are represented with atom features such as atom type, hybridization, charge, etc. While the edges are represented using bond features such as bond type, whether the bond is part of a ring etc. These atom and bond features are then used as an input to the message passing neural network to obtain the graph embeddings. The graph embeddings of olefins, ligands, and solvents are concatenated with other features to obtain the reaction representation of size 1544.

3. Implementation details

In this section, we discuss details of the implementation of various single-task and meta-learning methods used in this study.

3.1 Single-task methods

We have considered eight different single-task methods: random forest (RF), deep kernel learning (DKL), decision trees (DT), extreme gradient boosting (XGBoost), adaptive boosting (AdaBoost), ExtraTrees, support vector machine (SVM), and graph neural network (GNN). While all single-task methods utilize fingerprint-based molecular representation, GNN uses molecular graph as input. The hyperparameter tuning is performed on the validation set.

In case of RF, XGBoost, AdaBoost, Extratrees, the '*n_estimators*' hyperparameter is tuned and set to 200. All other hyperparameter values are set to their default setting, as they did not result in significance improvement in model performance. In case of DT and SVM, default hyperparameters are used.

For GNN, message passing neural network is used to learn the embeddings. Multiple message-passing steps are used to get the node representation. The edge network and gated recurrent unit are respectively used as message and update function. We use a set2set model in the readout step for global pooling over node vectors. The graph representation vector thus obtained is first passed through two fully connected neural network layers of dimension 1024 and 512, followed by an output layer. PReLU is used as the activation function and a dropout rate of 0.1 is applied to feed-forward NN layers. The Adam optimizer is used to train the model for 400 epochs with a learning rate of 0.001. The batch size is kept at 512.

For DKL, there are two important parts: a neural network as feature extractor and a Gaussian process (GP) for predictions. The 1544-dimensional fingerprint-based reaction representation (Figure S2) is first passed through two fully connected NN layers of dimension 1024 and 512. The reaction embedding thus obtained is used as an input to GP. A dropout rate of 0.1 is used with NN layers. PReLU is used as the activation function. The Matérn52 without automatic relevance determination is selected as the base kernel. The Adam optimizer with a learning rate of 0.001 is used to train the model for 400 epochs.

The training cost of all single-task methods in terms of wall-clock time is given in Table S1. The experiments are performed on a single NVIDIA RTX A5000. It can be seen that GNN is much slower than other single-task methods, while providing comparable performance to RF.

Table S1. Wall Clock Time Consumed in Training Single-Task Methods

	Method	Wall-clock time (in seconds)
1.	RF	16
2.	DT	3.5
3.	XGBoost	110
4.	AdaBoost	60
5.	ExtraTrees	24
6.	SVM	87
7.	DKL	55
8.	GNN	1324.7

3.2 Meta-learning methods

Three different meta-learning methods are considered: prototypical networks, Deep Kernel Transfer (DKT), and Adaptive Deep Kernel Fitting (ADKF). For all four meta-learning methods, we use 1544-dimensional fingerprint-based reaction feature as an input to the model (Figure S2). These are first passed through feed-forward NN with two layers of dimension 1024 and 512. The layers have a dropout rate of 0.1 and PReLU as activation function. All the model hyperparameters are tuned using the validation tasks. For meta-training, we use support set of size 512, while query set size is 64. A batch of five support sets are utilized to obtain the average validation loss on the corresponding query sets. We train the model for 1000 epochs and validate after every 50 epochs. Both ProtoNet and DKT use the Adam optimizer with a learning rate of 0.0001 and 0.001 respectively. For ProtoNet, "*Mahalanobis*" distance is chosen as the distance metric. The DKT and ADKF use Matérn52 without automatic relevance determination (ARD) as the base kernel. The kernel parameters of ADKF model are trained using the L-BFGS-B optimizer, while Adam optimizer with a learning rate of 0.0001is used to optimize the feature extractor parameters.

The meta-training and meta-testing cost of all meta-learning methods in terms of wall-clock time is given in Table S2. The experiments are performed on a single NVIDIA RTX A5000. Meta-training is a one-time process. The meta-testing costs of all meta-learning methods are significantly lower than single-task methods.

Table S2. Wall Clock Time Consumed in Meta-Training and Meta-Testing

	Methods	Wall-clock time (in seconds)	
		Meta-training	Meta-testing
1.	Prototypical networks	565	0.37
2.	DKT	540.9	0.37
3.	ADKF	2339.4	0.67
4.	ADKF-prior	2824	1.23

4. Model performance on random splits

In this section, we compare the model performance of different meta-learning and single-task methods on random splits. The performance is reported in terms of area under the precision-recall curve (AUPRC) as an average of ten random support-query splits. The error is standard deviation across ten runs. Additionally, the area under the receiver operating characteristic curve (AUROC) for all methods is also provided.

4.1 Performance of meta-learning method

The performance of prototypical networks, DKT, and ADKF in terms of AUPRC score is shown in Table S3. Five different support set sizes are considered: 8, 16, 32, 64, and 128. A query set size of 128 is used with all support set sizes.

Table S3. Model Performance of Prototypical Networks, DKT, and ADKF in Terms of AUROC and AUPRC Scores

Prototypical Networks		
Support set size	AUROC	AUPRC
8	0.6802 ± 0.0145	0.7431 ± 0.0153
16	0.7523 ± 0.0078	0.8092 ± 0.0102
32	0.7757 ± 0.0114	0.8442 ± 0.0081
64	0.8120 ± 0.0028	0.8661 ± 0.0043
128	0.8508 ± 0.0044	0.8971 ± 0.0035

DKT		
Support set size	AUROC	AUPRC
8	0.8109 ± 0.0074	0.8460 ± 0.0098
16	0.8329 ± 0.0063	0.8680 ± 0.0069
32	0.8540 ± 0.0042	0.8894 ± 0.0030
64	0.8637 ± 0.0037	0.8974 ± 0.0019
128	0.8732 ± 0.0010	0.9047 ± 0.0013
ADKF		
Support set size	AUROC	AUPRC
8	0.7305 ± 0.0117	0.8027 ± 0.0153
16	0.8189 ± 0.0044	0.8768 ± 0.0033
32	0.8457 ± 0.0051	0.8905 ± 0.0047
64	0.8535 ± 0.0021	0.8922 ± 0.0020
128	0.8744 ± 0.0080	0.9081 ± 0.0085

4.2 Performance of single-task methods

The performance of all single-task methods in terms of AUPRC and AUROC score is shown in Table S4. The performance of single-task methods (RF, GNN, and DKL) trained only on the support set of the test task is also reported. It can be noted that the single-task methods utilizing only the support set of the test task for training performs much worse than when trained on full training data $\mathcal{D}_{train}$.

Table S4. Model Performance of Single-task Methods in Terms of AUPRC and AUROC Scores

	RF	
Support set size	AUROC	AUPRC
8	0.5072 ± 0.0016	0.6479 ± 0.0009
16	0.5049 ± 0.0027	0.6437 ± 0.0011
32	0.5182 ± 0.0052	0.6534 ± 0.0030
64	0.5352 ± 0.0048	0.6625 ± 0.0077
128	0.5734 ± 0.0136	0.6832 ± 0.0128
$\mathcal{D}_{train}$	0.8120 ± 0.0050	0.8369 ± 0.0055
	GNN	
Support set size	AUROC	AUPRC
8	0.5120 ± 0.0070	0.6485 ± 0.0064
16	0.5278 ± 0.0045	0.6550 ± 0.0039
32	0.5244 ± 0.0047	0.6577 ± 0.0031
64	0.5535 ± 0.0079	0.6622 ± 0.0082

128	0.5834 ± 0.0143	0.6782 ± 0.0086
$\mathcal{D}_{train}$	0.8021 ± 0.0026	0.8259 ± 0.0021
	DKL	
Support set size	AUROC	AUPRC
8	0.4600 ± 0.0055	0.6290 ± 0.0026
16	0.4673 ± 0.0038	0.6369 ± 0.0040
32	0.4904 ± 0.0017	0.6432 ± 0.0015
64	0.4984 ± 0.0011	0.6388 ± 0.0028
128	0.4975 ± 0.0143	0.6531 ± 0.0037
$\mathcal{D}_{train}$	0.8364 ± 0.0114	0.8702 ± 0.0160
	DT	
Support set size	AUROC	AUPRC
$\mathcal{D}_{train}$	0.7253 ± 0.0017	0.7699 ± 0.0016
	XGBoost	
Support set size	AUROC	AUPRC
$\mathcal{D}_{train}$	0.7501 ± 0.0030	0.7811 ± 0.0023
	AdaBoost	
Support set size	AUROC	AUPRC
$\mathcal{D}_{train}$	0.6906 ± 0.0017	0.7460 ± 0.0014
	ExtraTrees	
Support set size	AUROC	AUPRC
$\mathcal{D}_{train}$	0.8135 ± 0.0038	0.8367 ± 0.0028
	SVM	
Support set size	AUROC	AUPRC
$\mathcal{D}_{train}$	0.7781 ± 0.0033	0.8017 ± 0.0020

5. Mathematical details of ADKF-prior

Let θ_{GP} and θ_{NN} denote the parameters of base kernel and feature extractor respectively. We consider task-specific parameters $\Psi_{adapt} = [\theta_{GP}, \theta_{NN}]$ to be the set of all parameters in a deep kernel. Since the size of support set is usually small and the feature extractor has a large number of parameters, some form of regularization is required for efficient learning while preventing overfitting in the inner loop. We incorporate this by employing a hierarchical Bayesian model and assign a Gaussian prior to the feature extractor parameters (Figure 6 in the main text). Thus,

$\theta_{NN} \sim \mathcal{N}(\theta_{NN}|\phi, \sigma^2 I)$ is normally distributed with mean ϕ and variance σ^2. The estimation of these prior parameters corresponds to meta-learning, with $\Psi_{meta} = [\phi, \sigma^2]$.

The task-specific parameters Ψ_{adapt} are estimated by minimizing the train loss $\mathcal{L}_T$ on the support set $\mathcal{S}_{\mathcal{T}}$, given the meta-learned parameters Ψ_{meta}:

$$\Psi^*_{\text{adapt}}(\Psi_{\text{meta}}, \mathcal{S}_{\mathcal{T}}) = \arg\min_{\Psi_{\text{adapt}}} \mathcal{L}_T(\Psi_{\text{adapt}}, \Psi_{\text{meta}}, \mathcal{S}_{\mathcal{T}}). \quad (1)$$

The train loss consists of negative log marginal likelihood and an additional term to incorporate the prior distribution over the feature extractor parameters:

$$\mathcal{L}_{\mathcal{T}} = -\log p\left(\mathcal{S}^y_{\mathcal{T}} | \mathcal{S}^{\mathbf{x}}_{\mathcal{T}}, \theta_{GP}, \theta_{NN}\right) - \log p\left(\theta_{NN}|\phi, \sigma^2\right). \quad (2)$$

The optimal task-specific parameters Ψ^*_{adapt} are obtained by solving the inner loop optimization problem with gradient-based methods.

It is to be noted that $\mathcal{L}_{\mathcal{T}}$ in Equation (2) corresponds to the train loss for a single task and allows individual task adaptation. Using Ψ^*_{adapt}, the meta-learned parameters Ψ_{meta} are estimated by minimizing the average predictive validation loss $\mathcal{L}_{\mathcal{V}}$ on the query set $\mathcal{Q}_{\mathcal{T}}$ of T randomly sampled training tasks (Equation (3)). The negative log predictive posterior is chosen as the validation loss $\mathcal{L}_{\mathcal{V}}$:

$$\Psi^*_{\text{meta}} = \arg\min_{\Psi_{\text{meta}}} \mathbb{E}_{\text{p}(\mathcal{T})}\left[\mathcal{L}_{\mathcal{V}}\left(\Psi_{\text{meta}}, \Psi^*_{\text{adapt}}(\Psi_{\text{meta}}, \mathcal{S}_{\mathcal{T}})\right)\right], \quad (3)$$

$$\mathcal{L}_{\mathcal{V}} = -\log \text{p}\left(\mathcal{Q}^y_{\mathcal{T}} \middle| \mathcal{Q}^{\mathbf{x}}_{\mathcal{T}}, \Psi^*_{\text{adapt}}(\Psi_{\text{meta}}, \mathcal{S}_{\mathcal{T}})\right). \quad (4)$$

During meta-testing, the predictive posterior distribution is used to make predictions on the test task $\mathcal{T}_*$ with the optimal parameters Ψ^*_{adapt} and Ψ^*_{meta} obtained after meta-training.

Computing the optimal meta-learned parameters Ψ^*_{meta} requires minimizing Equation (4). This corresponds to a bi-level optimization problem, that needs solving Equation (1) implicitly. One can use any gradient-based algorithm for the outer loop optimization (Equation (4)) by computing the total derivative of the validation loss $\mathcal{L}_{\mathcal{V}}$ with respect to the meta-parameters Ψ_{meta} (*hypergradient*):

$$\frac{\mathrm{d}\mathcal{L}_{\mathcal{V}}}{\mathrm{d}\Psi_{\mathrm{meta}}}=\frac{\partial\mathcal{L}_{\mathcal{V}}}{\partial\Psi_{\mathrm{meta}}}+\frac{\partial\mathcal{L}_{\mathcal{V}}}{\partial\Psi^{*}_{\mathrm{adapt}}}\frac{\partial\Psi^{*}_{\mathrm{adapt}}}{\partial\Psi_{\mathrm{meta}}}. \tag{5}$$

While the derivatives $\frac{\partial\mathcal{L}_{\mathcal{V}}}{\partial\Psi_{\mathrm{meta}}}$ and $\frac{\partial\mathcal{L}_{\mathcal{V}}}{\partial\Psi^{*}_{\mathrm{adapt}}}$ can in practice be obtained via auto-differentiation, computing $\frac{\partial\Psi^{*}_{\mathrm{adapt}}}{\partial\Psi_{\mathrm{meta}}}$ is the primary challenge. It necessitates backpropagation through the inner loop iterations (Equation (2)) and thus requires the path of optimization to be fully stored in memory. This process becomes increasingly memory intensive and computationally expensive with the number of gradient steps.

The Implicit Function Theorem (IFT) has been used for bilevel optimization problem encountered in meta-learning.[46,1] It provides a way to calculate the best response Jacobian $\frac{\partial\Psi^{*}_{\mathrm{adapt}}}{\partial\Psi_{\mathrm{meta}}}$ using the final result of the algorithm and doesn't need the path of the optimization. Thus, we use IFT to compute $\frac{\partial\Psi^{*}_{\mathrm{adapt}}}{\partial\Psi_{\mathrm{meta}}}$ given a task $\mathcal{T}'$ and meta-learned parameters Ψ'_{meta}:

$$\frac{\partial\Psi^{*}_{adapt}}{\partial\Psi_{meta}}|_{\Psi'_{meta}}=-\left(\frac{\partial^2\mathcal{L}_{T}}{\partial\Psi_{adapt}\,\partial\Psi^{T}_{adapt}}\right)^{-1}\frac{\partial^2\mathcal{L}_{T}}{\partial\Psi_{adapt}\,\partial\Psi^{T}_{meta}}|_{\Psi'_{meta},\Psi^{*}_{adapt}(\Psi'_{meta})} \tag{6}$$

An approximate solution to inner loop optimization ($\Psi^{*}_{\mathrm{adapt}}$) is considered, which can be obtained from any gradient-based optimizer, as discussed previously.

Although Equation (6) suggests a way of computing $\frac{\partial\Psi^{*}_{\mathrm{adapt}}}{\partial\Psi_{\mathrm{meta}}}$, the computation and inversion of the Hessian matrix $\frac{\partial^2\mathcal{L}_{T}}{\partial\Psi_{\mathrm{adapt}}\,\partial\Psi^{\mathrm{T}}_{\mathrm{adapt}}}$ can present difficulty. For instance, the inverse Hessian can be computed exactly if the number of parameters are less, as is the case with GP hyperparameters. But, inverting the Hessian can become computationally intractable for the large number of feature extractor parameters. However, there are several approaches to approximate the inverse Hessian.[2,3] Herein, we efficiently approximate the inverse Hessian with the Neumann series:

$$\left(\frac{\partial^2 \mathcal{L}_T}{\partial \Psi_{adapt}\, \partial \Psi_{adapt}^T}\right)^{-1} = \lim_{i\to\infty} \sum_{j=0}^{i} \left(I - \frac{\partial^2 \mathcal{L}_T}{\partial \Psi_{adapt}\, \partial \Psi_{adapt}}\right)^{j}. \tag{7}$$

The inverse Hessian is approximated with the first i terms in the Neumann series. This is done efficiently using the Jacobian-vector products.[4] Combining Equations (6) and (7), we get a tractable approximation for the hypergradient $\frac{d\mathcal{L}_V}{d\Psi_{meta}}$ (Equation (8)), which can then be used to update the parameters by gradient descent (section 5 in the Supporting Information).

$$\frac{d\mathcal{L}_V}{d\Psi_{meta}} = \frac{\partial \mathcal{L}_V}{\partial \Psi_{meta}} + \frac{\partial \mathcal{L}_V}{\partial \Psi_{adapt}^*}\left[-\sum_{0\le j\le i}\left(I - \frac{\partial^2 \mathcal{L}_T}{\partial \Psi_{adapt}\, \partial \Psi_{adapt}}\right)^{j}\right]\frac{\partial^2 \mathcal{L}_T}{\partial \Psi_{adapt}\, \partial \Psi_{meta}}. \tag{8}$$

6. Implementation of ADKF-prior

Here, we use 1544-dimensional fingerprint-based reaction feature as an input to the model. These are first passed through feed-forward NN with two layers of dimension 1024 and 512. The layers have a dropout rate of 0.1 and PReLU as activation function.

The inner loop optimization problem (Equation (1) in the main manuscript) is solved using the Adam optimizer. We use a learning rate of 0.01 for neural network parameters, θ_{NN}. While the base kernel parameters of GP, θ_{GP} are optimized using a learning rate of 0.1. The inner loop is run for a total of 40 epochs. For the outer loop optimization problem (Equation (3) in the main manuscript), the expected hypergradient is approximated by averaging the hypergradients of a batch of 5 randomly sampled train tasks. The meta-parameters are updated with the averaged hypergradient using Adam optimizer with a learning rate of 0.001 for 600 epochs. Matérn52 without automatic relevance determination (ARD) is chosen as the base kernel. We efficiently approximate the inverse Hessian with Neumann series (Equation (7) in the main manuscript). The inverse Hessian vector product $\frac{\partial \mathcal{L}_V}{\partial \Psi_{adapt}^*}\left[\left(\frac{\partial^2 \mathcal{L}_T}{\partial \Psi_{adapt}\, \partial \Psi_{adapt}^T}\right)^{-1}\right]$ is computed as shown in Algorithm 1. We use $i = 5$ and $\alpha = 0.0001$.

Algorithm 1 Neumann series approximation for the inverse Hessian vector product

Initialize p = v $\quad v = \frac{\partial \mathcal{L}_V}{\partial \Psi^*_{adapt}}$

for $j = 1 \ldots . i$ **do**

$\quad v -= \alpha \cdot \text{grad}\left(\frac{\partial^2 \mathcal{L}_T}{\partial \Psi_{adapt}}, \Psi_{adapt}, \text{grad\_outputs} = v\right)$

$\quad p += v$

return αp

7. Performance comparison of DKT and ADKF with ADKF-prior on random splits

The performance comparison ADKF-prior with DKT and ADKF on random splits in terms of AUPRC score is shown in Table S5. Six different support set sizes are considered: 5, 10, 15, 30, 64, and 128. A query set size of 128 is used with all support set sizes.

Table S5. Model Performance of DKT, ADKF, and ADKF-prior in Terms of AUROC and AUPRC Scores

DKT		
Support set size	AUROC	AUPRC
5	0.7377 ± 0.0159	0.7992 ± 0.0142
10	0.8164 ± 0.0148	0.8595 ± 0.0182
15	0.8219 ± 0.0101	0.8706 ± 0.0095
20	0.8349 ± 0.0024	0.8744 ± 0.0024
64	0.8637 ± 0.0037	0.8974 ± 0.0019
128	0.8732 ± 0.0010	0.9047 ± 0.0013
ADKF		
Support set size	AUROC	AUPRC
5	0.6732 ± 0.0210	0.7527 ± 0.0234
10	0.7756 ± 0.0246	0.8374 ± 0.0208
15	0.8263 ± 0.0088	0.8837 ± 0.0052
20	0.8434 ± 0.0054	0.8913 ± 0.0061
64	0.8535 ± 0.0021	0.8922 ± 0.0020
128	0.8744 ± 0.0080	0.9081 ± 0.0085
ADKF-prior		
Support set size	AUROC	AUPRC
5	0.7847 ± 0.0054	0.8459 ± 0.0030
10	0.8209 ± 0.0019	0.8766 ± 0.0017
15	0.8300 ± 0.0071	0.8871 ± 0.0045
20	0.8307 ± 0.0073	0.8907 ± 0.0043

64	0.8483 ± 0.0033	0.9052 ± 0.0029
128	0.8666 ± 0.0049	0.9110 ± 0.0033

8. Model performance on substrate-based splits

In this section, we compare the model performance of different meta-learning and single-task methods on substrate-based splits. The performance is reported in terms of area under the precision-recall curve (AUPRC) as an average of 30 random support-query splits. The error is reported in terms of standard deviation. Additionally, the area under the receiver operating characteristic curve (AUROC) for all methods is also provided.

8.1 Train-test split 1

8.1.1 Performance of meta-learning methods

The performance of prototypical networks, DKT, ADKF, and ADKF-prior in terms of AUPRC score is shown in Table S6. Five different support set sizes are considered: 8, 16, 32, 64, and 128. A query set size of 128 is used with all support set sizes.

Table S6. Model Performance of Prototypical Networks, DKT, ADKF, and ADKF-prior in Terms of AUROC and AUPRC Scores

Prototypical Networks		
Support set size	AUROC	AUPRC
8	0.6162 ± 0.0721	0.7663 ± 0.0598
16	0.6242 ± 0.0766	0.7872 ± 0.0561
32	0.6594 ± 0.0520	0.7984 ± 0.0523
64	0.6837 ± 0.0518	0.8202 ± 0.0400
128	0.7244 ± 0.0448	0.8433 ± 0.0378
DKT		
Support set size	AUROC	AUPRC
8	0.6995 ± 0.0935	0.8118 ± 0.0650
16	0.7078 ± 0.0638	0.8171 ± 0.0523
32	0.7241 ± 0.0517	0.8256 ± 0.0479
64	0.7155 ± 0.0716	0.8293 ± 0.0591
128	0.7329 ± 0.0467	0.8376 ± 0.0448
ADKF		
Support set size	AUROC	AUPRC
8	0.6434 ± 0.0938	0.7862 ± 0.0522

16	0.6836 ± 0.0806	0.8191 ± 0.0652
32	0.7126 ± 0.0490	0.8294 ± 0.0386
64	0.7344 ± 0.0572	0.8416 ± 0.0436
128	0.7511 ± 0.0528	0.8611 ± 0.0438
ADKF-prior		
Support set size	AUROC	AUPRC
8	0.7027 ± 0.0403	0.8221 ± 0.0329
16	0.7047 ± 0.0488	0.8274 ± 0.0463
32	0.7155 ± 0.0445	0.8322 ± 0.0415
64	0.7160 ± 0.0510	0.8411 ± 0.0436
128	0.7487 ± 0.0468	0.8591 ± 0.0464

8.1.2 Performance of single-task methods

The performance of single-task methods in terms of AUPRC and AUROC score is shown in Table S7. The performance is reported on full training data, $\mathcal{D}_{train}$ as well as $\mathcal{D}_{train}$+support set of test task. The support set sizes used are 8, 16, 32 ,64, and 128. It can be noted that adding the support set of the test task to $\mathcal{D}_{train}$ does not considerably change the performance of single-task methods, except DKL which shows an improvement with support set size of 32, 64, and 128.

Table S7. Model Performance of Single-task Methods in Terms of AUPRC and AUROC Scores

	RF	
Support set size	AUROC	AUPRC
$\mathcal{D}_{train}$	0.6626 ± 0.0371	0.7729 ± 0.0374
$\mathcal{D}_{train}$+8	0.5795 ± 0.0623	0.7186 ± 0.0433
$\mathcal{D}_{train}$+16	0.5696 ± 0.0490	0.7145 ± 0.0439
$\mathcal{D}_{train}$+32	0.5961 ± 0.0635	0.7162 ± 0.0388
$\mathcal{D}_{train}$+64	0.5851 ± 0.0431	0.7153 ± 0.0509
$\mathcal{D}_{train}$+128	0.6350 ± 0.0485	0.7367 ± 0.0543
	GNN	
Support set size	AUROC	AUPRC
$\mathcal{D}_{train}$	0.6508 ± 0.0042	0.7548 ± 0.0023
$\mathcal{D}_{train}$+8	0.6441 ± 0.0004	0.7510 ± 0.0021
$\mathcal{D}_{train}$+16	0.6494 ± 0.0054	0.7540 ± 0.0031
$\mathcal{D}_{train}$+32	0.6378 ± 0.0040	0.7475 ± 0.0022
$\mathcal{D}_{train}$+64	0.6351 ± 0.0046	0.7461 ± 0.0025
$\mathcal{D}_{train}$+128	0.6463 ± 0.0065	0.7525 ± 0.0039
	DKL	

Support set size	AUROC	AUPRC
$\mathcal{D}_{train}$	0.7022 ± 0.0468	0.8204 ± 0.0455
$\mathcal{D}_{train}$+8	0.6899 ± 0.0541	0.8070 ± 0.0463
$\mathcal{D}_{train}$+16	0.6949 ± 0.0381	0.8260 ± 0.0423
$\mathcal{D}_{train}$+32	0.7181 ± 0.0418	0.8322 ± 0.0432
$\mathcal{D}_{train}$+64	0.7233 ± 0.0589	0.8335 ± 0.0442
$\mathcal{D}_{train}$+128	0.7374 ± 0.0424	0.8381 ± 0.0450
	DT	
Support set size	AUROC	AUPRC
$\mathcal{D}_{train}$	0.6156 ± 0.0362	0.7386 ± 0.0327
$\mathcal{D}_{train}$+8	0.5516 ± 0.0574	0.7110 ± 0.0415
$\mathcal{D}_{train}$+16	0.5636 ± 0.0555	0.7100 ± 0.0428
$\mathcal{D}_{train}$+32	0.5824 ± 0.0514	0.7192 ± 0.0456
$\mathcal{D}_{train}$+64	0.6110 ± 0.0704	0.7374 ± 0.0517
$\mathcal{D}_{train}$+128	0.6215 ± 0.0389	0.7328 ± 0.0338
	XGBoost	
Support set size	AUROC	AUPRC
$\mathcal{D}_{train}$	0.6067 ± 0.0388	0.7316 ± 0.0361
$\mathcal{D}_{train}$+8	0.5633 ± 0.0480	0.7120 ± 0.0418
$\mathcal{D}_{train}$+16	0.5781 ± 0.0663	0.7278 ± 0.0578
$\mathcal{D}_{train}$+32	0.5758 ± 0.0589	0.7227 ± 0.0477
$\mathcal{D}_{train}$+64	0.5709 ± 0.0479	0.7172 ± 0.0361
$\mathcal{D}_{train}$+128	0.5603 ± 0.0271	0.6992 ± 0.0398
	AdaBoost	
Support set size	AUROC	AUPRC
$\mathcal{D}_{train}$	0.6039 ± 0.0428	0.7280 ± 0.0358
$\mathcal{D}_{train}$+8	0.5588 ± 0.0492	0.7074 ± 0.0430
$\mathcal{D}_{train}$+16	0.5637 ± 0.0542	0.7030 ± 0.0440
$\mathcal{D}_{train}$+32	0.5639 ± 0.0481	0.7020 ± 0.0355
$\mathcal{D}_{train}$+64	0.5939 ± 0.0426	0.7278 ± 0.0435
$\mathcal{D}_{train}$+128	0.6176 ± 0.0404	0.7310 ± 0.0364
	ExtraTrees	
Support set size	AUROC	AUPRC
$\mathcal{D}_{train}$	0.6463 ± 0.0492	0.7630 ± 0.0356
$\mathcal{D}_{train}$+8	0.6609 ± 0.0414	0.7602 ± 0.0453
$\mathcal{D}_{train}$+16	0.6527 ± 0.0455	0.7554 ± 0.0468
$\mathcal{D}_{train}$+32	0.6323 ± 0.0506	0.7421 ± 0.0408
$\mathcal{D}_{train}$+64	0.6670 ± 0.0430	0.7601 ± 0.0436
$\mathcal{D}_{train}$+128	0.6680 ± 0.0457	0.7641 ± 0.0458
	SVM	
Support set size	AUROC	AUPRC
$\mathcal{D}_{train}$	0.6345 ± 0.0392	0.7544 ± 0.0355

$\mathcal{D}_{train}$+8	0.6320 ± 0.0355	0.7438 ± 0.0385
$\mathcal{D}_{train}$+16	0.6412 ± 0.0360	0.7511 ± 0.0453
$\mathcal{D}_{train}$+32	0.6465 ± 0.0384	0.7538 ± 0.0365
$\mathcal{D}_{train}$+64	0.6476 ± 0.0389	0.7589 ± 0.0452
$\mathcal{D}_{train}$+128	0.6571 ± 0.0399	0.7618 ± 0.0373

8.1.3 Performance with different classification thresholds

We have also evaluated the performance of meta-models with different classification threshold. Two classification thresholds are used: 85 and 90. The results in terms of AUPRC and AUROC score are shown in Table S8.

Table S8. Model Performance of Meta-Learning and Single-Task Methods with Different Classification Thresholds

Classification threshold = 85		
	Prototypical networks	
Support set size	AUROC	AUPRC
8	0.6067 ± 0.0689	0.7198 ± 0.0546
16	0.6213 ± 0.0697	0.7338 ± 0.0654
32	0.6767 ± 0.0555	0.7652 ± 0.0572
64	0.6980 ± 0.0619	0.7926 ± 0.0688
128	0.7334 ± 0.0457	0.8189 ± 0.0403
	DKT	
Support set size	AUROC	AUPRC
8	0.7061 ± 0.1074	0.7746 ± 0.0818
16	0.7272 ± 0.0466	0.7918 ± 0.0472
32	0.7259 ± 0.0859	0.8117 ± 0.0582
64	0.7356 ± 0.0536	0.8139 ± 0.0512
128	0.7605 ± 0.0419	0.8259 ± 0.0436
	ADKF	
Support set size	AUROC	AUPRC
8	0.6761 ± 0.0554	0.7744 ± 0.0543
16	0.6898 ± 0.0596	0.7894 ± 0.0525
32	0.7192 ± 0.0459	0.8070 ± 0.0366
64	0.7394 ± 0.0415	0.8258 ± 0.0415
128	0.7759 ± 0.0397	0.8372 ± 0.0467
	ADKF-prior	
Support set size	AUROC	AUPRC
8	0.6909 ± 0.0504	0.7906 ± 0.0577
16	0.6979 ± 0.0471	0.7971 ± 0.0432
32	0.7017 ± 0.0418	0.8043 ± 0.0474
64	0.7224 ± 0.0424	0.8091 ± 0.0491

128	0.7500 ± 0.0369	0.8258 ± 0.0339
	RF	
Support set size	AUROC	AUPRC
$\mathcal{D}_{train}$	0.6874 ± 0.0385	0.7296 ± 0.0524
	DKL	
Support set size	AUROC	AUPRC
$\mathcal{D}_{train}$	0.6980 ± 0.0396	0.7988 ± 0.0462
Classification threshold = 90		
	Prototypical networks	
Support set size	AUROC	AUPRC
8	0.6156 ± 0.0682	0.6514 ± 0.0811
16	0.6292 ± 0.0771	0.6698 ± 0.0899
32	0.6534 ± 0.0586	0.6916 ± 0.0732
64	0.7012 ± 0.0464	0.7281 ± 0.0565
128	0.7293 ± 0.0420	0.7575 ± 0.0390
	DKT	
Support set size	AUROC	AUPRC
8	0.6727 ± 0.1570	0.7113 ± 0.1144
16	0.7308 ± 0.0684	0.7365 ± 0.0725
32	0.7337 ± 0.0533	0.7549 ± 0.0586
64	0.7489 ± 0.0484	0.7592 ± 0.0586
128	0.7468 ± 0.0410	0.7619 ± 0.0499
	ADKF	
Support set size	AUROC	AUPRC
8	0.6279 ± 0.0848	0.6770 ± 0.0885
16	0.6595 ± 0.0576	0.7065 ± 0.0650
32	0.6954 ± 0.0506	0.7259 ± 0.0601
64	0.7317 ± 0.0379	0.7511 ± 0.0509
128	0.7514 ± 0.0432	0.7721 ± 0.0569
	ADKF-prior	
Support set size	AUROC	AUPRC
8	0.6797 ± 0.0510	0.7103 ± 0.0567
16	0.6798 ± 0.0467	0.7117 ± 0.0523
32	0.6779 ± 0.0461	0.7176 ± 0.0510
64	0.7042 ± 0.0441	0.7415 ± 0.0400
128	0.7380 ± 0.0450	0.7719 ± 0.0516
	RF	
Support set size	AUROC	AUPRC
$\mathcal{D}_{train}$	0.7472 ± 0.0418	0.7243 ± 0.0500

	DKL	
Support set size	AUROC	AUPRC
$\mathcal{D}_{train}$	0.6903 ± 0.0431	0.7179 ± 0.0522

8.2 Train-test split 2

8.2.1 Performance of meta-learning methods

The performance of prototypical networks, DKT, ADKF, and ADKF-prior in terms of AUPRC score is shown in Table S9. Five different support set sizes are considered: 8, 16, 32, 64, and 128. A query set size of 128 is used with all support set sizes.

Table S9. Model Performance of Prototypical Networks, DKT, ADKF, and ADKF-prior in Terms of AUROC and AUPRC Scores

Prototypical Networks		
Support set size	AUROC	AUPRC
8	0.5870 ± 0.0819	0.7560 ± 0.0574
16	0.6136 ± 0.0701	0.7648 ± 0.0647
32	0.6527 ± 0.0631	0.7939 ± 0.0572
64	0.6985 ± 0.0545	0.8320 ± 0.0442
128	0.7542 ± 0.0414	0.8633 ± 0.0360
DKT		
Support set size	AUROC	AUPRC
8	0.6206 ± 0.1031	0.7712 ± 0.0829
16	0.6505 ± 0.0947	0.8029 ± 0.0644
32	0.6645 ± 0.0927	0.8164 ± 0.0645
64	0.6652 ± 0.0535	0.8223 ± 0.0527
128	0.7015 ± 0.0515	0.8353 ± 0.0322
ADKF		
Support set size	AUROC	AUPRC
8	0.6014 ± 0.0860	0.7680 ± 0.0660
16	0.6432 ± 0.0858	0.7854 ± 0.0653
32	0.6949 ± 0.0542	0.8200 ± 0.0495
64	0.7041 ± 0.0514	0.8323 ± 0.0407
128	0.7732 ± 0.0515	0.8693 ± 0.0475
ADKF-prior		
Support set size	AUROC	AUPRC
8	0.6635 ± 0.0384	0.8033 ± 0.0434
16	0.6849 ± 0.0560	0.8195 ± 0.0355
32	0.6795 ± 0.0538	0.8225 ± 0.0364
64	0.6995 ± 0.0379	0.8355 ± 0.0397

128	0.7344 ± 0.0432	0.8632 ± 0.0297

8.2.2 Performance of single-task methods

The performance of single-task methods in terms of AUPRC and AUROC score is shown in Table S10.

Table S10. Model Performance of Single-task Methods in Terms of AUPRC and AUROC Scores

	RF	
Support set size	AUROC	AUPRC
$\mathcal{D}_{train}$	0.6494 ± 0.0475	0.7437 ± 0.0420
	GNN	
Support set size	AUROC	AUPRC
$\mathcal{D}_{train}$	0.6242 ± 0.0038	0.7439 ± 0.0022
	DKL	
Support set size	AUROC	AUPRC
$\mathcal{D}_{train}$	0.6850 ± 0.0428	0.8177 ± 0.0523
	DT	
Support set size	AUROC	AUPRC
$\mathcal{D}_{train}$	0.5249 ± 0.0404	0.6912 ± 0.0380
	XGBoost	
Support set size	AUROC	AUPRC
$\mathcal{D}_{train}$	0.7760 ± 0.0471	0.7355 ± 0.0334
	AdaBoost	
Support set size	AUROC	AUPRC
$\mathcal{D}_{train}$	0.5965 ± 0.0513	0.7369 ± 0.0448
	ExtraTrees	
Support set size	AUROC	AUPRC
$\mathcal{D}_{train}$	0.6693 ± 0.0487	0.7680 ± 0.0426
	SVM	
Support set size	AUROC	AUPRC
$\mathcal{D}_{train}$	0.6508 ± 0.0396	0.7669 ± 0.0372

The data in Tables S9 and S10 is plotted in Figure S3. Only three single-task methods: RF, GNN, and DKL are shown for comparison with meta-learning methods.

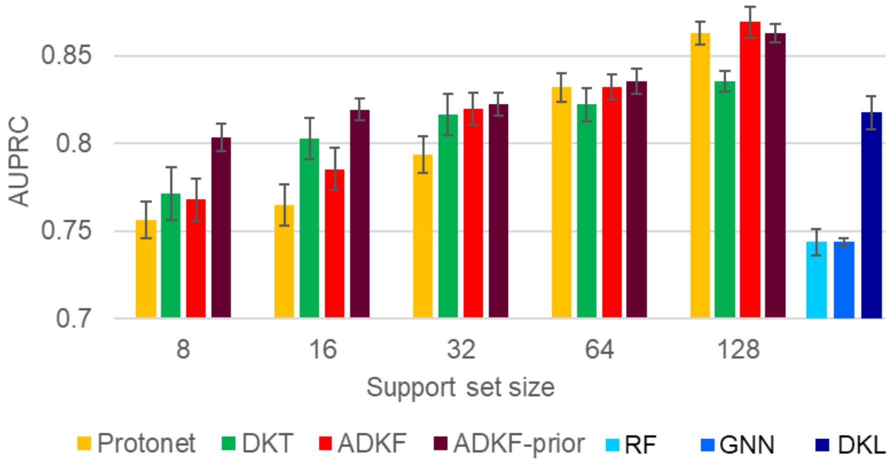

Figure S3. Summary of results for meta-learning and single-task methods on substrate-based train-test split2.

8.3 Train-test split 3

8.3.1 Performance of meta-learning methods

The performance of prototypical networks, DKT, ADKF, and ADKF-prior in terms of AUPRC score is shown in Table S11. Five different support set sizes are considered: 8, 16, 32, 64, and 128. A query set size of 128 is used with all support set sizes.

Table S11. Model Performance of Prototypical Networks, DKT, ADKF, and ADKF-prior in Terms of AUROC and AUPRC Scores

Prototypical Networks		
Support set size	AUROC	AUPRC
8	0.6117 ± 0.0764	0.7066 ± 0.0752
16	0.6415 ± 0.0623	0.7300 ± 0.0618
32	0.6523 ± 0.0515	0.7440 ± 0.0485
64	0.6908 ± 0.0491	0.7634 ± 0.0474
128	0.7272 ± 0.0468	0.7876 ± 0.0441
DKT		
Support set size	AUROC	AUPRC
8	0.6747 ± 0.0772	0.7449 ± 0.0716
16	0.6782 ± 0.0888	0.7564 ± 0.0626
32	0.6994 ± 0.0453	0.7712 ± 0.0521
64	0.7073 ± 0.0492	0.7759 ± 0.0525
128	0.7064 ± 0.0448	0.7838 ± 0.0514
ADKF		
Support set size	AUROC	AUPRC

8	0.6223 ± 0.0844	0.7212 ± 0.0748
16	0.6505 ± 0.0758	0.7415 ± 0.0721
32	0.6627 ± 0.0629	0.7529 ± 0.0509
64	0.6928 ± 0.0447	0.7596 ± 0.0507
128	0.7334 ± 0.0404	0.7922 ± 0.0433
ADKF-prior		
Support set size	AUROC	AUPRC
8	0.6793 ± 0.0528	0.7563 ± 0.0518
16	0.6878 ± 0.0434	0.7692 ± 0.0558
32	0.6697 ± 0.0400	0.7710 ± 0.0367
64	0.7068 ± 0.0438	0.7839 ± 0.0450
128	0.7357 ± 0.0439	0.8151 ± 0.0400

8.3.2 Performance of single-task methods

The performance of single-task methods in terms of AUPRC and AUROC score is shown in Table S12.

Table S12. Model Performance of Single-task Methods in Terms of AUPRC and AUROC Scores

	RF	
Support set size	AUROC	AUPRC
$\mathcal{D}_{train}$	0.6698 ± 0.0404	0.7118 ± 0.0440
	GNN	
Support set size	AUROC	AUPRC
$\mathcal{D}_{train}$	0.6535 ± 0.0028	0.6972 ± 0.0020
	DKL	
Support set size	AUROC	AUPRC
$\mathcal{D}_{train}$	0.6713 ± 0.0536	0.7575 ± 0.0621
	DT	
Support set size	AUROC	AUPRC
$\mathcal{D}_{train}$	0.5648 ± 0.0386	0.6555 ± 0.0453
	XGBoost	
Support set size	AUROC	AUPRC
$\mathcal{D}_{train}$	0.5992 ± 0.0330	0.6685 ± 0.0346
	AdaBoost	
Support set size	AUROC	AUPRC
$\mathcal{D}_{train}$	0.6077 ± 0.0469	0.6720 ± 0.0458
	ExtraTrees	
Support set size	AUROC	AUPRC

$\mathcal{D}_{train}$	0.6799 ± 0.0441	0.7211 ± 0.0441
	SVM	
Support set size	AUROC	AUPRC
$\mathcal{D}_{train}$	0.6401 ± 0.0416	0.7046 ± 0.0344

The data in Tables S11 and S12 is plotted in Figure S4. Only three single-task methods: RF, GNN, and DKL are shown for comparison with meta-learning methods.

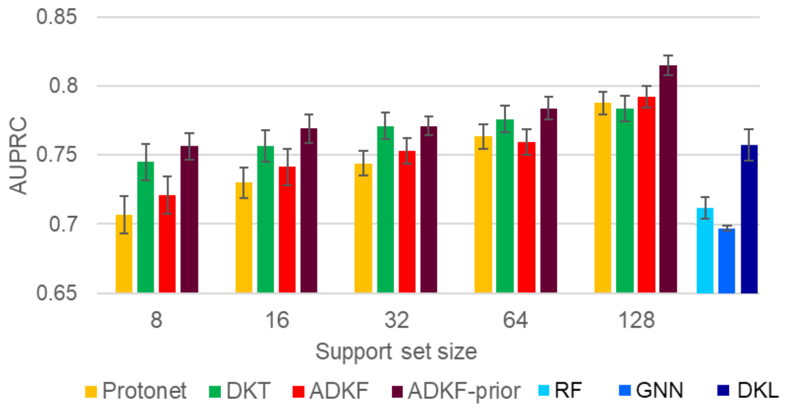

Figure S4. Summary of results for meta-learning and single-task methods on substrate-based train-test split 3.

8.4 Train-test split 4

8.4.1 Performance of meta-learning methods

The performance of prototypical networks, DKT, ADKF, and ADKF-prior in terms of AUPRC score is shown in Table S13. Five different support set sizes are considered: 8, 16, 32, 64, and 128. A query set size of 128 is used with all support set sizes.

Table S13. Model Performance of Prototypical Networks, DKT, ADKF, and ADKF-prior in Terms of AUROC and AUPRC Scores

Prototypical Networks		
Support set size	AUROC	AUPRC
8	0.6586 ± 0.0930	0.7640 ± 0.0741
16	0.7030 ± 0.0536	0.7862 ± 0.0569
32	0.7121 ± 0.0636	0.8099 ± 0.0531
64	0.7673 ± 0.0423	0.8357 ± 0.0439
128	0.7991 ± 0.0389	0.8630 ± 0.0317
DKT		

Support set size	AUROC	AUPRC
8	0.7289 ± 0.0963	0.8044 ± 0.0741
16	0.7679 ± 0.0581	0.8270 ± 0.0563
32	0.7770 ± 0.0390	0.8355 ± 0.0381
64	0.7707 ± 0.0421	0.8422 ± 0.0445
128	0.7833 ± 0.0403	0.8419 ± 0.0484
ADKF		
Support set size	AUROC	AUPRC
8	0.6525 ± 0.0818	0.7907 ± 0.0604
16	0.6885 ± 0.0646	0.8145 ± 0.0537
32	0.7205 ± 0.0595	0.8342 ± 0.0571
64	0.7288 ± 0.0567	0.8392 ± 0.0477
128	0.7690 ± 0.0638	0.8645 ± 0.0454
ADKF-prior		
Support set size	AUROC	AUPRC
8	0.6857 ± 0.0391	0.7727 ± 0.0494
16	0.6887 ± 0.0436	0.7735 ± 0.0460
32	0.7115 ± 0.0416	0.7967 ± 0.0421
64	0.7391 ± 0.0436	0.8242 ± 0.0458
128	0.7830 ± 0.0424	0.8561 ± 0.0370

8.4.2 Performance of single-task methods

The performance of single-task methods in terms of AUPRC and AUROC score is shown in Table S14.

Table S14. Model Performance of Single-task Methods in Terms of AUPRC and AUROC Scores

	RF	
Support set size	AUROC	AUPRC
$\mathcal{D}_{train}$	0.6772 ± 0.0340	0.7147 ± 0.0357
	GNN	
Support set size	AUROC	AUPRC
$\mathcal{D}_{train}$	0.6529 ± 0.0078	0.7009 ± 0.0049
	DKL	
Support set size	AUROC	AUPRC
$\mathcal{D}_{train}$	0.6978 ± 0.0494	0.7606 ± 0.0544
	DT	
Support set size	AUROC	AUPRC
$\mathcal{D}_{train}$	0.6155 ± 0.0420	0.6810 ± 0.0431

	XGBoost	
Support set size	AUROC	AUPRC
$\mathcal{D}_{train}$	0.5995 ± 0.0261	0.6628 ± 0.0399
	AdaBoost	
Support set size	AUROC	AUPRC
$\mathcal{D}_{train}$	0.6376 ± 0.0372	0.6751 ± 0.0513
	ExtraTrees	
Support set size	AUROC	AUPRC
$\mathcal{D}_{train}$	0.6758 ± 0.0479	0.7186 ± 0.0421
	SVM	
Support set size	AUROC	AUPRC
$\mathcal{D}_{train}$	0.7149 ± 0.0399	0.7387 ± 0.0445

The data in Tables S13 and S14 is plotted in Figure S5. Only three single-task methods: RF, GNN, and DKL are shown for comparison with meta-learning methods.

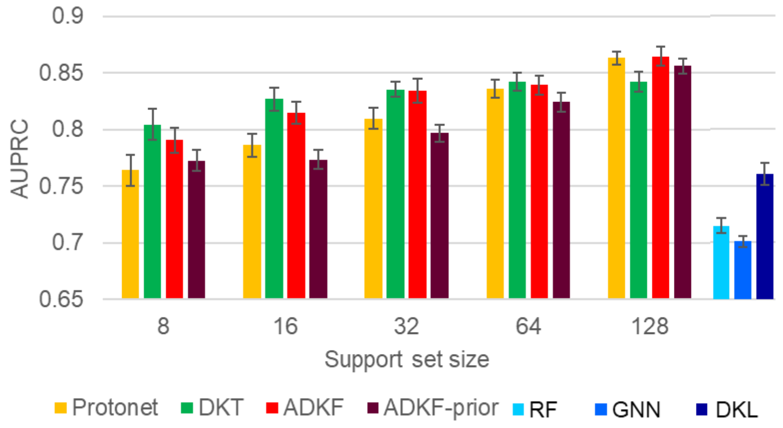

Figure S5. Summary of results for meta-learning and single-task methods on substrate-based train-test split 4.

8.5 Train-test split 5

8.5.1 Performance of meta-learning methods

The performance of prototypical networks, DKT, ADKF, and ADKF-prior in terms of AUPRC score is shown in Table S15. Five different support set sizes are considered: 8, 16, 32, 64, and 128. A query set size of 128 is used with all support set sizes.

Table S15. Model Performance of Prototypical Networks, DKT, ADKF, and ADKF-prior in Terms of AUROC and AUPRC Scores

Prototypical Networks		
Support set size	AUROC	AUPRC
8	0.5911 ± 0.0724	0.7147 ± 0.0532
16	0.6461 ± 0.0523	0.7513 ± 0.0486
32	0.6697 ± 0.0660	0.7662 ± 0.0509
64	0.7159 ± 0.0606	0.8128 ± 0.0499
128	0.7574 ± 0.0490	0.8335 ± 0.0465
DKT		
Support set size	AUROC	AUPRC
8	0.6519 ± 0.1039	0.7536 ± 0.0875
16	0.6725 ± 0.0694	0.7781 ± 0.0590
32	0.6915 ± 0.0650	0.7826 ± 0.0620
64	0.6923 ± 0.0505	0.8014 ± 0.0475
128	0.7049 ± 0.0436	0.8080 ± 0.0405
ADKF		
Support set size	AUROC	AUPRC
8	0.6435 ± 0.0804	0.7438 ± 0.0721
16	0.6754 ± 0.0634	0.7676 ± 0.0625
32	0.6876 ± 0.0768	0.7795 ± 0.0672
64	0.7333 ± 0.0491	0.8100 ± 0.0599
128	0.7558 ± 0.0404	0.8345 ± 0.0322
ADKF-prior		
Support set size	AUROC	AUPRC
8	0.6817 ± 0.0502	0.7803 ± 0.0488
16	0.6870 ± 0.0397	0.7933 ± 0.0332
32	0.7035 ± 0.0379	0.8075 ± 0.0428
64	0.7176 ± 0.0578	0.8217 ± 0.0456
128	0.7440 ± 0.0473	0.8350 ± 0.0472

8.5.2 Performance of single-task methods

The performance of single-task methods in terms of AUPRC and AUROC score is shown in Table S16.

Table S16. Model Performance of Single-task Methods in Terms of AUPRC and AUROC Scores

	RF	
Support set size	AUROC	AUPRC

$\mathcal{D}_{train}$	0.6338 ± 0.0309	0.7003 ± 0.0422
	GNN	
Support set size	AUROC	AUPRC
$\mathcal{D}_{train}$	0.6433 ± 0.0055	0.7099 ± 0.0035
	DKL	
Support set size	AUROC	AUPRC
$\mathcal{D}_{train}$	0.6695 ± 0.0424	0.7727 ± 0.0542
	DT	
Support set size	AUROC	AUPRC
$\mathcal{D}_{train}$	0.6251 ± 0.0458	0.6998 ± 0.0465
	XGBoost	
Support set size	AUROC	AUPRC
$\mathcal{D}_{train}$	0.5849 ± 0.0287	0.6679 ± 0.0444
	AdaBoost	
Support set size	AUROC	AUPRC
$\mathcal{D}_{train}$	0.6383 ± 0.0349	0.6974 ± 0.0387
	ExtraTrees	
Support set size	AUROC	AUPRC
$\mathcal{D}_{train}$	0.6259 ± 0.0293	0.6976 ± 0.0382
	SVM	
Support set size	AUROC	AUPRC
$\mathcal{D}_{train}$	0.6172 ± 0.0314	0.6892 ± 0.0425

The data in Tables S15 and S16 is plotted in Figure S6. Only three single-task methods: RF, GNN, and DKL are shown for comparison with meta-learning methods.

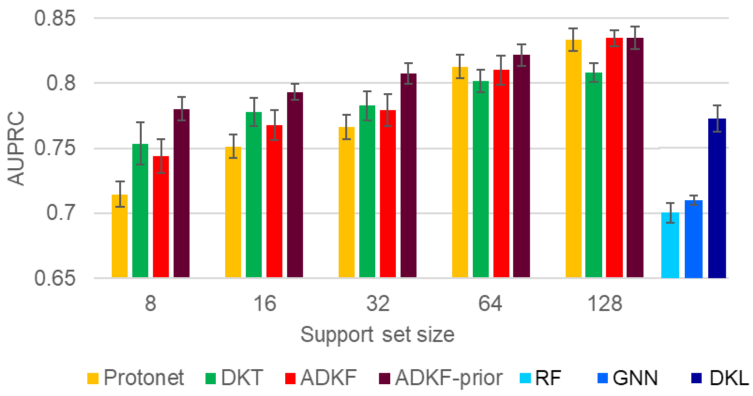

Figure S6. Summary of results for meta-learning and single-task methods on substrate-based train-test split 5.

8.6 Train-test split 6

8.6.1 Performance of meta-learning methods

The performance of prototypical networks, DKT, ADKF, and ADKF-prior in terms of AUPRC score is shown in Table S17. Five different support set sizes are considered: 8, 16, 32, 64, and 128. A query set size of 128 is used with all support set sizes.

Table S17. Model Performance of Prototypical Networks, DKT, ADKF, and ADKF-prior in Terms of AUROC and AUPRC Scores

Prototypical Networks		
Support set size	AUROC	AUPRC
8	0.5974 ± 0.0929	0.7216 ± 0.0831
16	0.6207 ± 0.0569	0.7346 ± 0.0590
32	0.6434 ± 0.0830	0.7663 ± 0.0649
64	0.7007 ± 0.0476	0.7920 ± 0.0499
128	0.7362 ± 0.0440	0.8224 ± 0.0358
DKT		
Support set size	AUROC	AUPRC
8	0.6610 ± 0.0765	0.7526 ± 0.0685
16	0.6756 ± 0.0536	0.7675 ± 0.0610
32	0.6913 ± 0.0477	0.7716 ± 0.0622
64	0.6999 ± 0.0409	0.7872 ± 0.0450
128	0.7136 ± 0.0414	0.7989 ± 0.0370
ADKF		
Support set size	AUROC	AUPRC
8	0.5827 ± 0.0938	0.7085 ± 0.0668
16	0.6251 ± 0.0898	0.7444 ± 0.0782
32	0.6720 ± 0.0521	0.7589 ± 0.0521
64	0.6927 ± 0.0597	0.7792 ± 0.0521
128	0.7221 ± 0.0509	0.8012 ± 0.0548
ADKF-prior		
Support set size	AUROC	AUPRC
8	0.6633 ± 0.0600	0.7637 ± 0.0567
16	0.6766 ± 0.0434	0.7701 ± 0.0584
32	0.6850 ± 0.0513	0.7835 ± 0.0539
64	0.7031 ± 0.0461	0.7987 ± 0.0544
128	0.7324 ± 0.0403	0.8167 ± 0.0398

8.6.2 Performance of single-task methods

The performance of single-task methods in terms of AUPRC and AUROC score is shown in Table S18.

Table S18. Model Performance of Single-task Methods in Terms of AUPRC and AUROC Scores

	RF	
Support set size	AUROC	AUPRC
$\mathcal{D}_{train}$	0.6339 ± 0.0445	0.6973 ± 0.0505
	GNN	
Support set size	AUROC	AUPRC
$\mathcal{D}_{train}$	0.6344 ± 0.0054	0.6967 ± 0.0031
	DKL	
Support set size	AUROC	AUPRC
$\mathcal{D}_{train}$	0.6606 ± 0.0444	0.7641 ± 0.0433
	DT	
Support set size	AUROC	AUPRC
$\mathcal{D}_{train}$	0.5759 ± 0.0303	0.6726 ± 0.0467
	XGBoost	
Support set size	AUROC	AUPRC
$\mathcal{D}_{train}$	0.5726 ± 0.0322	0.6532 ± 0.0380
	AdaBoost	
Support set size	AUROC	AUPRC
$\mathcal{D}_{train}$	0.6001 ± 0.0414	0.6849 ± 0.0395
	ExtraTrees	
Support set size	AUROC	AUPRC
$\mathcal{D}_{train}$	0.6383 ± 0.0395	0.6941 ± 0.0457
	SVM	
Support set size	AUROC	AUPRC
$\mathcal{D}_{train}$	0.6141 ± 0.0392	0.6888 ± 0.0467

The data in Tables S17 and S18 is plotted in Figure S7. Only three single-task methods: RF, GNN, and DKL are shown for comparison with meta-learning methods.

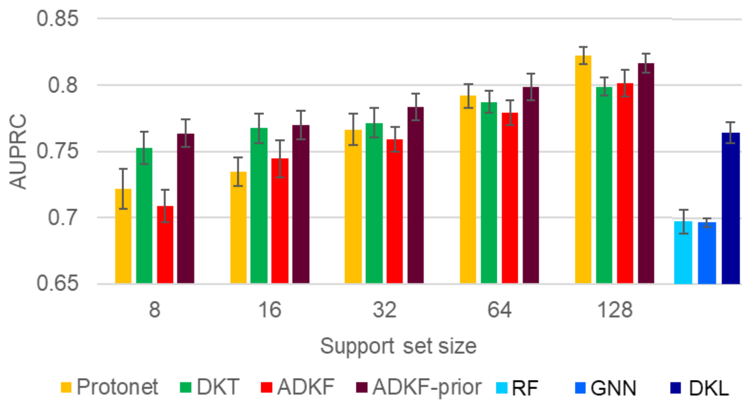

Figure S7. Summary of results for meta-learning and single-task methods on substrate-based train-test split 6.

9. Performance on time-based splits

A set of 245 Ir- and Rh-catalyzed AHO reaction which were not present in the original dataset are used as an out-of-sample test set. The identity of these reactions is shown in Scheme S1.

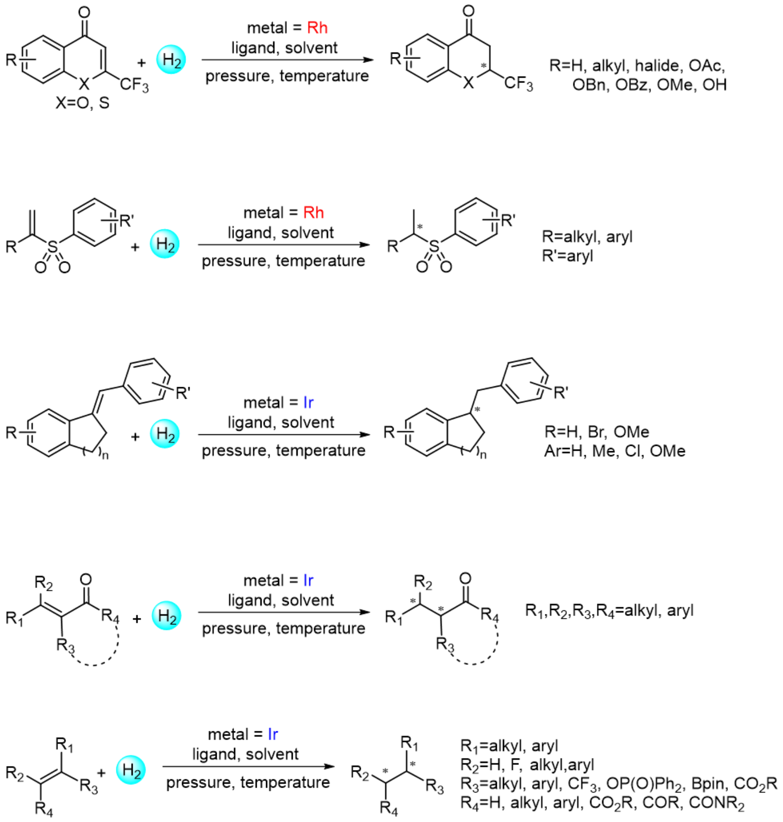

Scheme S1. A general representation of reactions utilized as the out-of-sample test set.

The model generalizability is evaluated on the out-of-sample test. The performance averaged over 10 random support-query splits of the test tasks is reported in terms of AUPRC

and AUROC scores. Table S19 presents the results of all four meta-learning methods on the out-of-sample test set.

Table S19. Model Performance of Prototypical Networks, DKT, ADKF, and ADKF-prior in Terms of AUROC and AUPRC Scores

DKT		
Support set size	AUROC	AUPRC
8	0.5981 ± 0.0426	0.8475 ± 0.0278
16	0.6497 ± 0.0390	0.8579 ± 0.0171
32	0.6596 ± 0.0273	0.8801 ± 0.0141
64	0.6678 ± 0.0156	0.8900 ± 0.0040
ADKF		
Support set size	AUROC	AUPRC
8	0.6276 ± 0.0190	0.8542 ± 0.0027
16	0.6724 ± 0.0864	0.8834 ± 0.0332
32	0.7650 ± 0.0309	0.9191 ± 0.0162
64	0.7937 ± 0.0140	0.9364 ± 0.0035
ADKF-prior		
Support set size	AUROC	AUPRC
8	0.7451 ± 0.0308	0.9062 ± 0.0221
16	0.7679 ± 0.0112	0.9252 ± 0.0062
32	0.7725 ± 0.0122	0.9037 ± 0.0096
64	0.8245 ± 0.0308	0.9434 ± 0.0107
Prototypical Networks		
Support set size	AUROC	AUPRC
8	0.5925 ± 0.0359	0.8491 ± 0.0146
16	0.6316 ± 0.0110	0.8591 ± 0.0059
32	0.7520 ± 0.0235	0.9200 ± 0.0119
64	0.7780 ± 0.0135	0.9225 ± 0.0049

In Table S20, we compare the model performance of single-task methods with meta-learning methods (shown in Table S19). In addition to different support set sizes of the test task, we also give the model performance of single-task methods trained on $\mathcal{D}_{train}$. It can be noted from Table S20 that the performance of single-task methods with a support set size of 64 is comparable and sometimes better than $\mathcal{D}_{train}$. This can be due to a support set more similar to the query set of the test task in the earlier case.

Table S20. Model Performance of Prototypical Networks, DKT, ADKF, and ADKF-prior in Terms of AUROC and AUPRC Scores

RF		
Support set size	AUROC	AUPRC
8	0.4920 +- 0.0129	0.7941 +- 0.0176
16	0.5614 +- 0.0194	0.8316 +- 0.0126
32	0.5874 +- 0.0259	0.8495 +- 0.0180
64	0.6473 +- 0.0317	0.8604 +- 0.0139
$\mathcal{D}_{train}$	0.6347 +- 0.0157	0.8504 +- 0.0077
GNN		
Support set size	AUROC	AUPRC
8	0.5189 +- 0.0145	0.8148 +- 0.0115
16	0.5696 +- 0.0121	0.8396 +- 0.0070
32	0.5840 +- 0.0390	0.8356 +- 0.0169
64	0.7147 +- 0.0170	0.8764 +- 0.0090
$\mathcal{D}_{train}$	0.6395 +- 0.0115	0.8558 +- 0.0042
DKL		
Support set size	AUROC	AUPRC
8	0.5300 +- 0.0263	0.8206 +- 0.0116
16	0.5213 +- 0.0419	0.8024 +- 0.0225
32	0.5785 +- 0.0277	0.8400 +- 0.0160
64	0.6747 +- 0.0167	0.8767 +- 0.0127
$\mathcal{D}_{train}$	0.6542 +- 0.0156	0.8782 +- 0.0150

10. References

(1) A. Rajeswaran, C. Finn, S. M. Kakade, S. Levine, *Advances in neural information processing systems*, **2019**, *32*.

(2) J. Lorraine, P. Vicol, D. Duvenaud, In *International conference on artificial intelligence and statistics* **2020**, 1540-1552.

(3) R. M. Clarke, E. T. Oldewage, J. M. Hernández-Lobato, In *International Conference on Learning Representations* **2022**.

(4) B. A. Pearlmutter, *Neural computation*, **1994**, *6*, 147-160.
